# A complexity transition in displaced Gaussian Boson sampling

**DOI:** 10.1038/s41534-025-01062-5

**Published:** 2025-07-09

**Authors:** Zhenghao Li, Naomi R. Solomons, Jacob F. F. Bulmer, Raj B. Patel, Ian A. Walmsley

**Affiliations:** 1 Department of Physics, Imperial College London, London, UK; 2 Quantum Engineering Centre for Doctoral Training, Centre for Nanoscience and Quantum Information, University of Bristol, Bristol, UK; 3 Quantum Engineering Technology Labs, H. H. Wills Physics Laboratory and Department of Electrical and Electronic Engineering, University of Bristol, Bristol, UK; 4 Present Address: LIP6 CNRS, Sorbonne Université, Paris, France

**Keywords:** Quantum optics, Computational science, Quantum information

## Abstract

Gaussian Boson Sampling (GBS) is the problem of sampling from the output of photon-number-resolving measurements of squeezed states input to a linear optical interferometer. For purposes of demonstrating quantum computational advantage as well as practical applications, a large photon number is often desirable. However, producing squeezed states with high photon numbers is experimentally challenging. In this work, we examine the computational complexity implications of increasing the photon number by introducing coherent states. This displaces the state in phase space and as such we call this modified problem *Displaced GBS*. By utilising a connection to the matching polynomial in graph theory, we first describe an efficient classical algorithm for Displaced GBS when displacement is high or when the output state is represented by a non-negative graph. Then we provide complexity theoretic arguments for the quantum advantage of the problem in the low-displacement regime and numerically quantify where the complexity transition occurs.

## Introduction

An important milestone in quantum computing is the demonstration of quantum advantage, the goal of performing a task on a quantum device while providing unequivocal evidence that the same task cannot be performed on a classical device within a reasonable amount of time. Advances in the coherent control of large quantum systems have led to experiments that are difficult or perhaps even intractable for conventional computing methods to emulate^1–7^.

Gaussian Boson Sampling (GBS), as a variant of the Boson Sampling problem, has emerged as a route to demonstrating quantum advantage that is particularly suited to near-term optical implementations^8–11^. Here, one measures the photon number statistics of a Gaussian state, prepared by passing squeezed states through a Haar random, multi-mode interferometer. Plausible conjectures in complexity theory provide reasonable evidence for the classical hardness of the sampling task that is further supported by algorithmic studies^11,12^. Impressive experimental implementations of the scheme have already been demonstrated on the scale of 100−255 photons^1–4^. They represent major milestones towards demonstrating quantum advantage, while also stimulating the development of new classical algorithms that have significantly raised the bar for exponential quantum advantage in GBS^12,13^.

A major challenge faced by these experiments is scaling. The largest experimental demonstrations of GBS in refs. ^1–4^ have so far only utilised squeezed vacuum states as input to the interferometer. Preparing squeezed vacuum states with large mean number of photons is experimentally challenging and also sensitive to loss. This poses an obstacle to further scaling up the total photon number in these experiments.

Coherent states are an under-explored resource in the context of GBS. They are incorporated in the original theoretical framework^9,10^, but their capability has not yet been exploited. This class of states presents a means by which the total number of photons in the system can be easily increased, as they are readily generated by lasers and their amplitude and phase can be easily controlled using conventional optical components. They are also necessary for certain practical applications that map onto the GBS problem, such as molecular vibronic spectra simulations^14–18^ and graph similarity measurements^19^. Importantly, the preparation of coherent states is tolerant to loss, as a coherent state with photon loss remains a coherent state, albeit with a smaller amplitude.

In contrast to squeezed states, coherent states input into a passive linear-optical networks do not generate entanglement at the output, and a GBS problem that only takes coherent states as input can be exactly solved efficiently on a classical computer^20^. Therefore, adding coherent states to a GBS problem might seem to make the problem more ‘classical’, diluting the quantum character of the output state. Despite this, a small-scale experimental demonstration of GBS using both squeezed and coherent light has shown a clear distinction in its output photon distribution from a fully classical model, even when there is a considerable ‘classical’ component to the state^21^.

In this work, we analyse the computational complexity of the *‘Displaced GBS’* (D-GBS) problem. The name derives from the effect of coherent light on the phase-space distribution of the optical quantum state, which is to displace it by an amount proportional to the amplitude of the coherent state. The sampling probability function, which is given by the loop-Hafnian function^10,22^, is connected to the matching polynomial that is widely studied in graph theory^23–26^. In particular, we propose a *Uniform D-GBS* scheme that takes identical displaced squeezed states as input to a Haar random interferometer and analyse its average-case complexity.

In complexity theory, one way to prove that a problem is ‘easy’ is by designing an efficient classical algorithm to solve it. Since quantum advantage is usually assumed to be an exponential separation in running time, we consider an algorithm to be efficient as long as its running time is sub-exponential. Based on the Taylor approximation method with quasi-polynomial runtime by Barvinok^25^, we describe a new loop-Hafnian estimation algorithm for a D-GBS problem that is either displacement dominant or when it encodes a non-negative weighted graph.

Second, to show that the D-GBS problem can still be ‘hard’ in certain regimes, we apply complexity-theoretic arguments. We prove the worst-case complexity for estimating the loop-Hafnian at non-zero displacement, which rules out exact solutions to D-GBS by efficient classical methods. To study the approximate hardness of the problem, we derive the matrix distribution in Uniform D-GBS, over which the loop-Hafnians are evaluated. We conjecture that when displacement is low, this distribution leaves little structure to separate the worst- and average-case complexity of the loop-Hafnian, while also not concentrating the loop-Hafnian values around specific values. We provide evidence for the conjectures, and by using a standard technique developed in refs. ^8,11,27^, we prove, up to the conjectures, that there is a regime where no efficient classical algorithm is expected to solve the Uniform D-GBS problem even approximately. Numerically, we also provide evidence for how the average-case complexity of estimating the loop-Hafnian changes upon adding more displacement, based on the Taylor approximation method.

A second major obstacle to experimentally implementing GBS is photon loss. A recently developed tensor network algorithm has challenged the quantum advantage claims of the latest GBS experiments by exploiting their high levels of loss^28^. While the addition of displacement cannot directly mitigate the effects of loss—indeed, adding displacement to an already lossy GBS implementation does not make it harder to simulate using the method of ref. ^28^— it also does not exacerbate them. Despite adding more ‘classical’ light, the addition of displacement does not lower the loss-threshold for the simulability of GBS, while the preparation of coherent states themselves is loss-tolerant, as lost photons in a coherent state can be compensated by preparing it with a larger amplitude. Comparing regular GBS to D-GBS, our work shows a regime where the latter can retain the former’s quantum advantage without significantly adding to the experimental difficulty all while presenting further advantages in achieving large photon numbers and mapping to practical applications.

## Results

### D-GBS and the matching polynomial

**Fig. 1:**
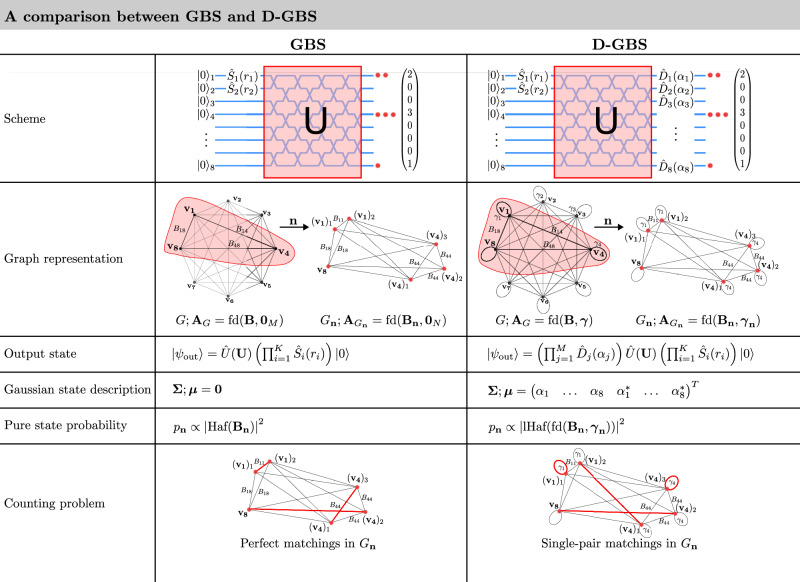
A comparison between GBS and D-GBS with *M* = 8 modes and *K* = 2 squeezed vacuum states. The interferometer is described by the unitary matrix ***U*** and multi-mode operator $\hat{U}({\boldsymbol{U}})$. An example detection sample of ${\boldsymbol{n}}={\left(2\,0\,0\,3\,0\,0\,0\,1\right)}^{T}$ is shown. In D-GBS, the problem is described by a complex symmetric matrix fd(***B***, ***γ***), which defines an undirected, weighted graph, *G*. The edges are weighted by *B*_*ij*_, while the loop-weights by *γ*_*k*_. The detection of ***n*** selects a subgraph and repeats the *i*-th vertex by *n*_*i*_ times to construct *G*_***n***_. Repeated vertices are connected to each other by the diagonal terms of the ***B*** matrix, *B*_*ii*_. The probability of sampling ***n*** is proportional to $\left\vert {\rm{lHaf}}{\left({\rm{fd}}\left({{\boldsymbol{B}}}_{{\boldsymbol{n}}},{{\boldsymbol{\gamma }}}_{{\boldsymbol{n}}}\right)\right\vert }^{2}\right.$, which sums over the single-pair matchings of *G*_***n***_. An example of a single-pair matching is highlighted in red. In the absence of displacement, ***γ*** = **0**, the graph reduces to a loopless graph. The loop-Hafnian becomes the Hafnian, ${\rm{lHaf}}\left({\rm{fd}}({{\boldsymbol{B}}}_{{\boldsymbol{n}}},{{\bf{0}}}_{N})={\rm{Haf}}({{\boldsymbol{B}}}_{{\boldsymbol{n}}})\right.$, which sums over the perfect matchings of *G*_***n***_^9,10^, an example of which also highlighted in red.

The D-GBS problem can be visualised as the sampling of subgraphs from a graph representation of the Gaussian state, as shown in Fig. 1. Compared to regular non-displaced GBS, the addition of displacement introduces loops to the graph, which are edges that connect vertices to themselves. We will first describe this graph representation before connecting it to the matching polynomial that is widely studied in graph theory.

Any *M*-mode Gaussian state can be uniquely represented by a 2*M* × 2*M* covariance matrix, ***Σ***, and a length-2*M* vector of means, ***μ***. Measuring all *M* modes with photon-number-resolving detectors (PNRDs) generates a sample $${\boldsymbol{n}}={\left(\begin{array}{rcl}{n}_{1}&\ldots &{n}_{M}\end{array}\right)}^{T}$$. The total photon number is denoted as $N=\mathop{\sum }\nolimits_{i = 1}^{M}{n}_{i}$. In the absence of loss and noise, the pure-state probability of generating sample ***n*** is^10,22^:

1 $${p}_{{\boldsymbol{n}}}=\frac{\exp \left(-\frac{1}{2}{{\boldsymbol{\mu }}}^{\dagger }{{\boldsymbol{\Sigma }}}_{Q}^{-1}{\boldsymbol{\mu }}\right)}{\sqrt{\det ({{\boldsymbol{\Sigma }}}_{Q})}\mathop{\prod }\nolimits_{j = 1}^{M}{n}_{j}!}\times {\left\vert {\rm{lHaf}}\left({\rm{fd}}({{\boldsymbol{B}}}_{{\boldsymbol{n}}},{{\boldsymbol{\gamma }}}_{{\boldsymbol{n}}})\right)\right\vert }^{2}.$$

where ***Σ***_*Q*_ is the covariance matrix of Q function, the lHaf(⋅) denotes the loop-Hafnian function, and the symbol fd(⋅) denotes replacing the diagonal of matrix ***B***_***n***_ with the vector ***γ***_***n***_. In the rest of this paper, we will occasionally drop the fd(⋅) for notation simplicity but its use is implicit in the loop-Hafnian function.

The complex symmetric matrix ***B*** and vector ***γ*** are constructed from ***Σ*** and ***μ***, and the subscript ***n*** denotes repeating the *i*-th row and column of ***B*** (or the *i*-th element of ***γ***) by *n*_*i*_ times. The *N* × *N* matrix fd(***B***_***n***_, ***γ***_***n***_) defines the adjacency matrix of an undirected, complex-weighted graph, denoted *G*_***n***_. The loop-Hafnian function, lHaf(***B***_***n***_, ***γ***_***n***_), is defined as^10,22,29^

2 $${\rm{lHaf}}({{\boldsymbol{B}}}_{{\boldsymbol{n}}},{{\boldsymbol{\gamma}}}_{{\boldsymbol{n}}})=\mathop{\sum}\limits_{\Phi \in {\rm{SPM}}({G}_{{\boldsymbol{n}}})}\mathop{\prod}\limits_{{(i,j)}{\in \Phi}\atop{i\ne j}}({{\boldsymbol{B}}}_{{\boldsymbol{n}}})_{ij}\mathop{\prod}\limits_{(k,k)\in \Phi}({{{{\gamma }}}_{{\boldsymbol{n}}}})_{k}$$

where SPM(*G*_***n***_) denotes the set of all *single-pair matchings* on *G*_***n***_. A single pair matching is a subset of edges, including loops, that is incident on every vertex *once and only once*. A loop is considered to only connect to its incident vertex once.

In this paper, we primarily study D-GBS where every mode has a non-zero displacement, which results in non-zero loop weights on every vertex of the graph representation. In this case, we can define a new matrix $\tilde{{\boldsymbol{B}}}$ by rewriting the loop-Hafnian as the same function but on a matrix of diagonal of ones:

3 $${\rm{lHaf}}({{\boldsymbol{B}}}_{{\boldsymbol{n}}},{{\boldsymbol{\gamma }}}_{{\boldsymbol{n}}})=\left(\mathop{\prod }\limits_{i=1}^{M}{{\boldsymbol{\gamma }}}_{i}^{{n}_{i}}\right){\rm{lHaf}}({\tilde{{\boldsymbol{B}}}}_{{\boldsymbol{n}}},{{\bf{1}}}_{N}),$$

where **1**_*N*_ is a length-*N* vector $${{\bf{1}}}_{N}={\left(\begin{array}{lll}1&\ldots &1\end{array}\right)}^{T}$$, the matrix $\tilde{{\boldsymbol{B}}}$ is constructed by

4 $${\tilde{B}}_{ij}=\frac{{B}_{ij}}{{\gamma }_{i}{\gamma }_{j}},$$

and the subscript ***n*** denotes the same repetition of rows and columns.

The inclusion of loops widens the range of graph problems that map to the quantum sampling problem. Furthermore, the problem of counting single-pair matchings in D-GBS is exactly the monomer-dimer problem in statistical physics^23^, and Equation (3) allows for the loop-Hafnian to be rewritten as the matching polynomial in graph theory, which enjoys a long line of research in computational complexity^24–26,30,31^.

On a complex-weighted *loopless* graph $\tilde{G}$ with an *N* × *N* adjacency matrix $\tilde{{\boldsymbol{A}}}$, the matching polynomial is defined as

5 $$g(z;\tilde{{\boldsymbol{A}}})=\sum _{\Theta \in {\mathcal{M}}(\tilde{G})}\left(\mathop{\prod}\limits_{(i,j)\in \Theta }{\tilde{A}}_{ij}\right){z}^{| \Theta | },$$

where ${\mathcal{M}}(\tilde{G})$ is the set of all *matchings* on $\tilde{G}$. A matching, *Θ*, is a subset of edges that is incident on every vertex *at most once*. The cardinality ∣*Θ*∣ is the number of edges inside *Θ*, which is upper bounded by $| \Theta | \le \lfloor \frac{N}{2}\rfloor$. As such, the maximum degree of the matching polynomial is $\lfloor \frac{N}{2}\rfloor$.

**Fig. 2:**
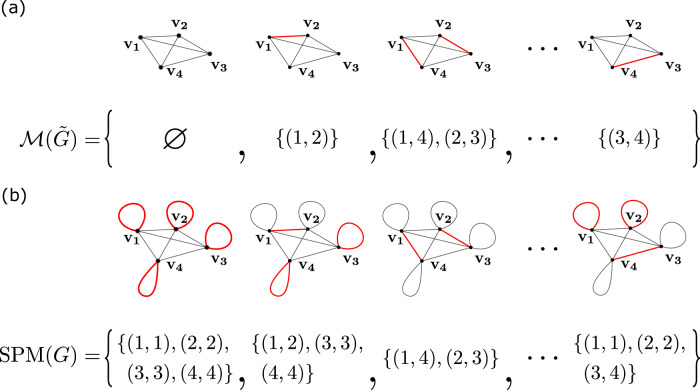
Matchings and single-pair matchings. **a** Enumerating the matchings on some graph $\tilde{G}$ without loops. **b** If loops are added to every vertex of $\tilde{G}$, every single-pair matching on the updated graph *G* has a one-to-one mapping to a unique matching in $\tilde{G}$. If all loops in *G* are weighted by one, then the loop-Hafnian on *G* is equivalent to the matching polynomial on $\tilde{G}$.

If we add a loop of weight one to every vertex in $\tilde{G}$, to construct a new graph *G* with adjacency matrix ${\rm{fd}}(\tilde{{\boldsymbol{A}}},{{\bf{1}}}_{N})$, then it is easy to see that summing over the matchings of $\tilde{G}$ is equivalent to summing over the single-pair matchings of *G*. Every matching, *Θ*, in graph $\tilde{G}$ maps to a unique single-pair matching, Φ, in graph *G* by connecting the vertices outside the matching to themselves via loops. This is illustrated with an example in Fig. 2a, b. In other words, the matching polynomial $g(z;\tilde{A})$ can be equivalently defined as:

6 $$g(z;\tilde{{\boldsymbol{A}}})={\rm{lHaf}}(z\tilde{{\boldsymbol{A}}},{{\bf{1}}}_{N}).$$

To put this in the context of D-GBS, evaluation of Equation (3) can be rewritten as evaluating a matching polynomial at *z* = 1:

7 $${\rm{lHaf}}({\tilde{{\boldsymbol{B}}}}_{{\boldsymbol{n}}},{{\bf{1}}}_{N})=g(1;{\tilde{{\boldsymbol{B}}}}_{{\boldsymbol{n}}}).$$

Multiplicative-error approximation of the matching polynomial is a #P-hard problem^24,26^. By Equation (6), multiplicative-error approximation of the loop-Hafnian would then also be #P-hard. We define *multiplicative-error* approximation as follows: an estimator *q* approximates some value *p* to within multiplicative-error *η* if ∣*q* − *p*∣ ≤ *η*∣*p*∣. On the other hand, an *additive-error* approximation is defined as giving an estimator *q*, such that ∣*q* − *p*∣ ≤ *ϵ*, where *ϵ* is the additive error.

The complexity class of a problem is a statement of its worst-case complexity. There can, however, be classes of ${\tilde{{\boldsymbol{B}}}}_{{\boldsymbol{n}}}$ instances with a special structure that renders the loop-Hafnian, ${\rm{lHaf}}\left({\tilde{{\boldsymbol{B}}}}_{{\boldsymbol{n}}},{{\bf{1}}}_{N}\right)$, efficient to estimate. In later sections we will see such complexity transitions for two special structures in the $\tilde{{\boldsymbol{B}}}$ matrix: when its off-diagonal magnitudes are small and when its entries are non-negative.

### Hiding in uniform D-GBS

For the general D-GBS problem, the matrix $\tilde{{\boldsymbol{B}}}$ in Equation (3) can be constructed to be any arbitrary symmetric matrix by suitable choice of squeezing, displacement and interferometer parameters^32–34^. In this section, we propose a scheme, named *Uniform D-GBS*, following the ensuing requirements. The submatrices ${\tilde{{\boldsymbol{B}}}}_{{\boldsymbol{n}}}$ will then ‘hide’ a distribution related to independent and identically distributed (i.i.d.) Gaussian random variables, over which we will study the average-case complexity of the loop-Hafnian. We will discuss how to relax these constraints in an experimental implementation in the Methods section.

1. The input states are *K* identical displaced squeezed vacuum states, $\mathop{\prod }\nolimits_{j = 1}^{K}{\hat{D}}_{j}(\beta ){\hat{S}}_{j}(r)| 0\left.\right\rangle$, with displacement parameter *β* and squeezing parameter *r*;
2. The *M*-mode interferometer is characterised by a Haar random unitary matrix ***U***;
3. The number of non-vacuum input modes is upper-bounded by $K\le \sqrt{M}$; and
4. The total mean photon number is set at one photon per non-vacuum input mode: $\overline{N}=K$, such that $| \beta {| }^{2}+{\sinh }^{2}r=1$.

**Fig. 3:**
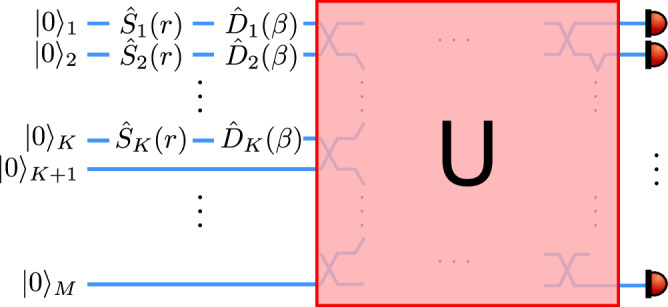
Uniform D-GBS example. Identical displaced squeezed states are input into *K* input modes. The input states are interfered on an *M*-mode interferometer with *M* ≥ *K*^2^ and is characterised by a Haar random unitary ***U***. After the interferometer the output photons are measured by PNRDs.

An illustration of the scheme is shown in Fig. 3. By Condition 1, the pair (***B***_***n***_, ***γ***_***n***_) for some sampling outcome ***n*** is derived to be:

8 $${{\boldsymbol{B}}}_{{\boldsymbol{n}}}=\tanh (r){{\boldsymbol{U}}}_{{\boldsymbol{n}},{{\bf{1}}}_{K}}{{\boldsymbol{U}}}_{{\boldsymbol{n}},{{\bf{1}}}_{K}}^{T},$$

9 $${{\boldsymbol{\gamma }}}_{{\boldsymbol{n}}}=({\beta }^{* }-\beta \tanh (r)){{\boldsymbol{U}}}_{{\boldsymbol{n}},{{\bf{1}}}_{K}}{{\bf{1}}}_{N},$$

where, without loss of generality, the squeezing parameter *r* is assumed to be positive real, as its phase can be absorbed into the interferometer. The matrix ${{\boldsymbol{U}}}_{{\boldsymbol{n}},{{\bf{1}}}_{K}}$ is constructed by repeating the *i*-th row of ***U*** by *n*_*i*_ times and only keeping the first *K* columns. Matrix multiplication with the column vector **1**_*N*_ returns sums of each row. More explicitly, this is:

10 $${\gamma}_{i}=({\beta }^{*}-\beta \tanh (r))\mathop{\sum}\limits_{j = 1}^{K}{U}_{ij}.$$

Since ***U*** is Haar random, in general we have *γ*_*i*_ ≠ 0 for all *i*.

Equations (8) and (9) allow us to separate the squeezing and displacement parameters from the interferometer transfer matrix. We go one step further and define a complex parameter *w*:

11 $$w=\frac{{\beta }^{* }-\beta \tanh (r)}{\sqrt{\tanh (r)}}.$$

The magnitude of *w* characterises the ratio of displacement to squeezing in the Uniform D-GBS scheme. For a fixed *w*, one can solve for the values of *r* and *β* using Eq. (11) and Condition 4.

Using Eqs. (8), (9) and (11), the probability of sampling ***n*** can be rewritten as

12 $${p}_{{\boldsymbol{n}}}\propto {\left|{\rm{lHaf}}\left({{\boldsymbol{U}}}_{{\boldsymbol{n}},{{\bf{1}}}_{K}}{{\boldsymbol{U}}}_{{\boldsymbol{n}},{{\bf{1}}}_{K}}^{T},w{{\boldsymbol{U}}}_{{\boldsymbol{n}},{{\bf{1}}}_{K}}{{\bf{1}}}_{N}\right)\right|}^{2}$$

where *w* becomes a scaling factor on the diagonal of the loop-Hafnian. In other words, changing the displacement and squeezing in Uniform D-GBS only changes the relative weights of the diagonal and off-diagonal elements in the loop-Hafnian. The distribution of these elements are determined by the matrices ${{\boldsymbol{U}}}_{{\boldsymbol{n}},{{\bf{1}}}_{K}}$.

Reference ^8^ showed that, when $\overline{N}\le \sqrt{M}$, the likelihood of collision events, i.e. detecting more than one photon in any output mode, is small – this is the ‘collision-less’ condition. This means we can treat ${{\boldsymbol{U}}}_{{\boldsymbol{n}},{{\bf{1}}}_{K}}$ as an *N* × *K* random submatrix of some Haar random ***U***.

For output samples with total photon number upper-bounded by $N\le \sqrt{M}$, which is a substantial subset of all outcomes given Condition 4, Ref. ^8^ further conjectured that the submatrix ${{\boldsymbol{U}}}_{{\boldsymbol{n}},{{\bf{1}}}_{K}}$ is approximately an independent and identically distributed (i.i.d.) complex Gaussian matrix ***X*** with mean zero and variance 1/*M*, denoted as

13 $${{\boldsymbol{U}}}_{{\boldsymbol{n}},{{\bf{1}}}_{K}} \sim {\boldsymbol{X}}\in {{\mathcal{G}}}_{N,K}(0,1/M).$$

This is the ‘hiding’ conjecture.

Together, the collision-less condition and the hiding conjecture give the loop-Hafnian function in Eq. (12) an underlying Gaussian distribution. Using the same loop-Hafnian rescaling technique in Eq. (3), we can rescale this loop-Hafnian to be:

14 $$\begin{array}{ll}{\rm{lHaf}}\left({{\boldsymbol{XX}}}^{T},w{\boldsymbol{X}}{{\bf{1}}}_{N}\right)\\=\left(\mathop{\prod }\limits_{i = 1}^{M}{({{\boldsymbol{\gamma }}}_{i})}^{{n}_{i}}\right){\rm{lHaf}}\left(\frac{1}{{w}^{2}}\tilde{{\boldsymbol{X}}},{{\bf{1}}}_{N}\right)\\=\left(\mathop{\prod }\limits_{i = 1}^{M}{({{\boldsymbol{\gamma }}}_{i})}^{{n}_{i}}\right)g\left(\frac{1}{{w}^{2}};\tilde{{\boldsymbol{X}}}\right),\end{array}$$

where matrix $\tilde{{\boldsymbol{X}}}$ is constructed from i.i.d. Gaussians ${\boldsymbol{X}}\in {{\mathcal{G}}}_{N,K}(0,1/M)$:

15 $${\tilde{X}}_{ij}=\frac{{({{\boldsymbol{XX}}}^{T})}_{ij}}{{\left({\boldsymbol{X}}{{\bf{1}}}_{N}\right)}_{i}{\left({\boldsymbol{X}}{{\bf{1}}}_{N}\right)}_{j}}.$$

The distribution of $\tilde{{\boldsymbol{X}}}$ is preserved if the Gaussian distribution is rescaled to ${\boldsymbol{X}}\in {{\mathcal{G}}}_{N,K}(0,1)$. We denote the matrix ensemble defined in Equation (15) as $\tilde{{\boldsymbol{X}}}\in {\tilde{{\mathcal{G}}}}_{N,K}(0)$.

### Diagonally-dominant loop-Hafnians in general D-GBS

Physically, the D-GBS problem is expected to become simulable when there is a large amount of displacement relative to squeezing^21^. Mathematically, this corresponds to a small $| {\tilde{B}}_{ij}|$ for $\tilde{{\boldsymbol{B}}}$ matrix in Eq. (3), or a large ∣*w*∣ for the *w* parameter in Eq. (14), both of which result in a diagonally-dominant matrix on which the loop-Hafnian is calculated.

In this special case, the loop-Hafnian can be approximated in quasi-polynomial time by the Taylor approximation method developed in refs. ^25,30,35–37^. The method is applicable to any polynomial that is non-zero for some domain in the complex plane and works by truncating the Taylor expansion of its logarithm in that domain.

More specifically, for an *N* × *N* matrix $\tilde{{\boldsymbol{A}}}$, if there is some disc in the complex plane with radius *R* > 0 such that the matching polynomial $g(z;\tilde{{\boldsymbol{A}}})\,\ne\, 0$ for all ∣*z*∣ < *R*, then inside the non-zero disc the Taylor truncation can approximate $\ln g(z;\tilde{{\boldsymbol{A}}})$ to within arbitrary additive error *ϵ* > 0 and can be computed in quasi-polynomial time ${N}^{O(\ln N-\ln \epsilon )}$. The detailed derivations are given in the 'Methods' section. If one can approximate $\ln g(z)$ to any additive error *ϵ* > 0, then one can approximate $g(z;\tilde{{\boldsymbol{A}}})$ to any multiplicative error *η* = *e*^*ϵ*^ − 1.

To establish a connection with diagonally-dominant loop-Hafnians, let’s consider an *N* × *N* matrix $\tilde{{\boldsymbol{A}}}$ that satisfies $|{\tilde{A}}_{ij}|\,\ll\,1$, for all *i*, *j*. Using Equation (6), the loop-Hafnian, ${\rm{lHaf}}(\tilde{{\boldsymbol{A}}},{{\bf{1}}}_{N})$, can be expressed as:

16 $$\begin{array}{ll}{\rm{lHaf}}(\tilde{{\boldsymbol{A}}},{{\bf{1}}}_{N})=1+{\mathop{\sum}\limits_{\{(i,j)\}\in\mathcal{M}}}{\tilde{A}}_{ij}+{\mathop{\sum}\limits_{\{(i,j),(k,l)\}\in\mathcal{M}}}{\tilde{A}}_{ij}{\tilde{A}}_{kl}+\ldots \end{array}$$

If the magnitude of the matrix elements, $| {\tilde{A}}_{ij}|$, are sufficiently small, the magnitude of the higher-order terms decay to zero and ${\rm{lHaf}}(\tilde{{\boldsymbol{A}}},{{\bf{1}}}_{N})$ is non-zero.

Now suppose we can find a bound 0 < *ζ* < 1, such that Eq. (16) is non-zero if $| {\tilde{A}}_{ij}| < \zeta$ for all *i*, *j*. In order to estimate ${\rm{lHaf}}(\tilde{{\boldsymbol{A}}},{{\bf{1}}}_{N})$, we can fix a real number *λ* satisfying 0 < *λ* < 1, such that $| {\tilde{A}}_{ij}| \le \lambda \zeta$ for all *i*, *j*. Then, the matching polynomial $g(z;\tilde{{\boldsymbol{A}}})$ is non-zero for any ∣*z*∣ < 1/*λ*. This enables the Taylor approximation method to approximate the matching polynomial at *z* = 1, which is precisely the loop-Hafnian value we wanted to estimate: ${\rm{lHaf}}(\tilde{{\boldsymbol{A}}},{{\bf{1}}}_{N})=g(1;\tilde{{\boldsymbol{A}}})$. The associated runtime is ${N}^{{O}_{\lambda }(\ln N-\ln \epsilon )}$, where the subscript in the big-*O* notation denotes that the implied constant depends only on *λ*.

The outstanding task is therefore to identify the bound on $| {\tilde{A}}_{ij}|$, such that ${\rm{lHaf}}(\tilde{{\boldsymbol{A}}},{{\bf{1}}}_{N})$ is non-zero and can be efficiently estimated. We prove Theorems 1 and 2.

#### Theorem 1

For an *N* × *N* complex symmetric matrix $\tilde{{\boldsymbol{A}}}$ that satisfies

17 $$| {\tilde{A}}_{ij}| < \frac{1}{{\rm{e}}(2N-3)}\,{\rm{for}}\,{\rm{all}}\,{\rm{i}}\,\ne\, {\rm{j}},$$

given any *ϵ* > 0, the function ${\rm{lHaf}}(\tilde{{\boldsymbol{A}}},{{\bf{1}}}_{N})$ can be approximated to within multiplicative error *ϵ* in time ${N}^{O(\ln N-\ln \epsilon )}$.

#### Theorem 2

For an *N* × *N* complex symmetric matrix $\tilde{{\boldsymbol{A}}}$ that satisfies

18 $$\sum _{j\ne i}| {\tilde{A}}_{ij}| < \frac{1}{N-1}\,\,\text{for all}\,\,i\in [1,N],$$

given any *ϵ* > 0, the function ${\rm{lHaf}}(\tilde{{\boldsymbol{A}}},{{\bf{1}}}_{N})$ can be approximated to within multiplicative error *ϵ* in time ${N}^{O(\ln N-\ln \epsilon )}$.

The proof for Theorem 1 uses results from refs. ^38,39^, and the proof for Theorem 2 adapts a method from ref. ^37^. The details of both proofs are provided in the Supplemental MaterialsSI. Equation (18) is typically less applicable than Eq. (17), except for a special matrix $\tilde{{\boldsymbol{A}}}$ where a row might have one off-diagonal element that violates latter, but the sums of matrix elements still satisfy the former.

For a general D-GBS problem, if matrix ${\tilde{{\boldsymbol{B}}}}_{{\boldsymbol{n}}}$ in Eq. (3) satisfies either condition, then ${\rm{lHaf}}({\tilde{{\boldsymbol{B}}}}_{{\boldsymbol{n}}},{{\bf{1}}}_{N})$ can be estimated within quasi-polynomial time, and one can approximately sample from the target distribution in Eq. (1) using a chain rule method^40^. The probability of these conditions being satisfied increases as the magnitude $\left\vert {\tilde{B}}_{ij}\right\vert$ decreases, which corresponds to a higher ratio of displacement to squeezing, as shown in the Methods section. However, the required ratio could be very significant, if ${\tilde{{\boldsymbol{B}}}}_{{\boldsymbol{n}}}$ were to satisfy either condition deterministically for *all* outcomes ***n***.

### Diagonally-dominant loop-Hafnians in uniform D-GBS

If we allow the algorithm to fail for a fraction of outcomes ***n***, the requirement on displacement can be made less stringent. For this, we consider the underlying distribution of ${\tilde{{\boldsymbol{B}}}}_{{\boldsymbol{n}}} \sim \frac{1}{{w}^{2}}\tilde{{\boldsymbol{X}}}$ for $\tilde{{\boldsymbol{X}}}\in {\tilde{{\mathcal{G}}}}_{N,K}(0)$ in our proposed *Uniform D-GBS* scheme, where *N* is the total photon number in outcome ***n*** and *K* is the number of non-vacuum input modes. We use the hiding conjecture and consider where the loop-Hafnian, ${\rm{lHaf}}\left(\frac{1}{{w}^{2}}\tilde{{\boldsymbol{X}}},{{\bf{1}}}_{N}\right)$, or equivalently the matching polynomial, $g\left(\frac{1}{{w}^{2}};\tilde{{\boldsymbol{X}}}\right)$, is *on average* efficient to estimate.

**Fig. 4:**
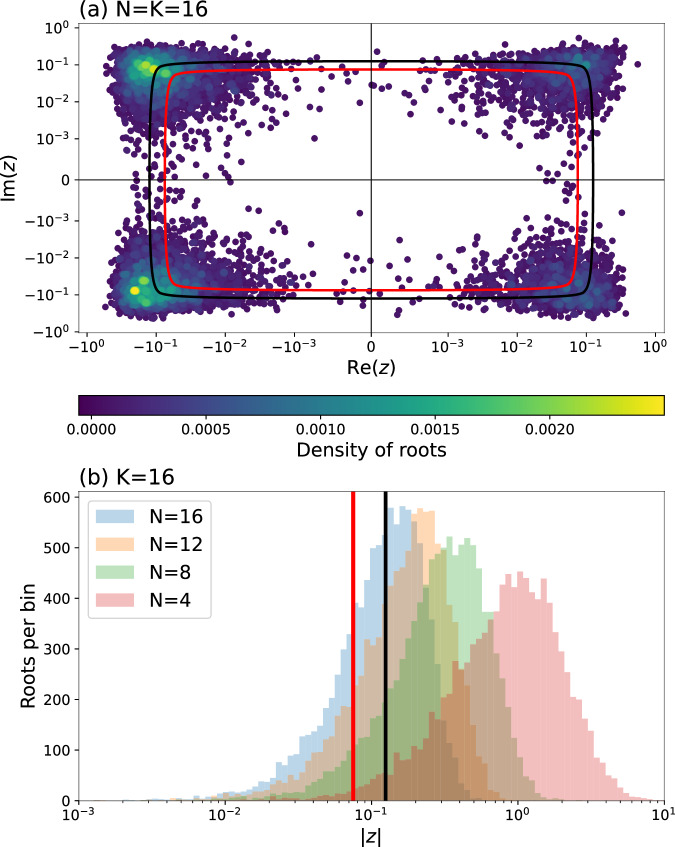
The distribution of zeroes of the matching polynomial. **a** The distribution of the smallest-magnitude roots of $g(z;\tilde{{\boldsymbol{X}}})$ for 10,000 $\tilde{{\boldsymbol{X}}}$ matrices drawn from ${\tilde{{\mathcal{G}}}}_{16,16}(0)$ (Eq. (15)). There is a region about the origin of the complex plane where the roots are less dense. The black box encompasses 50% of all plotted roots and the red box 25%. In other words, the matching polynomial $g(z;\tilde{{\boldsymbol{X}}})$ is empirically non-zero with 50% (75%) probability if the complex number *z* is inside the black (red) box. **b** The magnitude distribution of the smallest-magnitude roots of $g(z;\tilde{{\boldsymbol{X}}})$ for *N* ≤ *K* = 16. The magnitudes are higher for smaller *N*. The black and red lines correspond to the black and red boxes in (**a**), and the line-widths equal the error bars for 95% confidence level. In the Uniform D-GBS scheme, *N* is the total number of photons and *K* is the number of non-vacuum input modes.

We approach the problem numerically. Fig. 4(a), (b) plot the distribution of zeroes of $g(z;\tilde{{\boldsymbol{X}}})$ for $\tilde{{\boldsymbol{X}}}\in {\tilde{{\mathcal{G}}}}_{N,16}(0)$ for *N* ≤ 16. Clearly, there is a region near the origin of the complex plane where the zeroes are less dense, and the area of this region increases with decreasing *N*. The larger ∣*w*∣ is, the more likely it is that, for a random $\tilde{{\boldsymbol{X}}}$, the matching polynomial satisfies $g(z;\tilde{{\boldsymbol{X}}})\ne 0$ for $| z| \le \frac{1}{| w{| }^{2}}$. The Taylor approximation method can then approximate this to arbitrary multiplicative error.

**Fig. 5:**
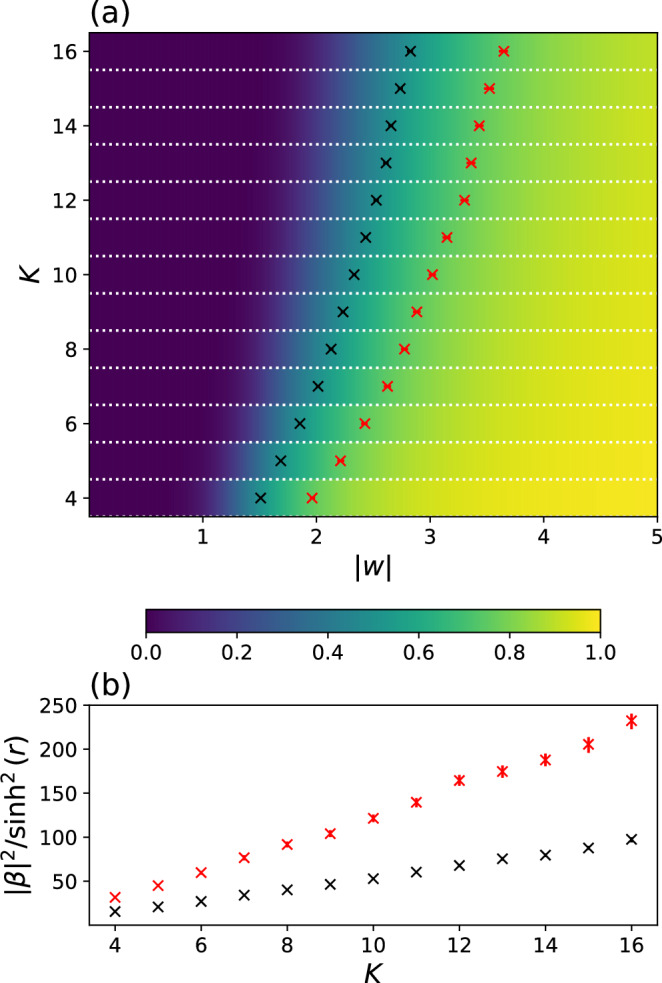
Efficient estimation of the matching polynomial. **a** Colour plot of the probability that $g(z;\tilde{{\boldsymbol{X}}})\ne 0$ for $| z| < \frac{1}{| w{| }^{2}}$ for different values of ∣*w*∣ and $\tilde{{\boldsymbol{X}}}\in {\tilde{{\mathcal{G}}}}_{K,K}(0)$. Probabilities estimated by bootstrapping from 10,000 randomly drawn $\tilde{{\boldsymbol{X}}}$ matrices. Black crosses indicate 50% probability, and red 75%. Error bars are included for 95% confidence level, though for many points not visible on this scale. **b** The photon number ratio $\frac{| \beta {| }^{2}}{{\sinh }^{2}(r)}$ calculated from Equation (11) for the *w* values corresponding to the black and red crosses in (**a**).

The lack of zeroes near the origin of the complex plane is true for all $\tilde{{\boldsymbol{X}}}\in {\tilde{{\mathcal{G}}}}_{N\le K,K}(0)$ up to *K* = 16, whose matching polynomials we numerically computed. Fig. 5(a) plots the probability that $g(z;\tilde{{\boldsymbol{X}}})\,\ne\, 0$ for $| z| < \frac{1}{| w{| }^{2}}$ and *N* = *K* as a colour plot. The black crosses indicate 50% probability and the red 75%. The probabilities would be even higher for *N* < *K*, as illustrated by Fig. 4(b). Given *w*, by solving Eq. (11) under Condition 4, we can solve for the displacement parameter *β* and squeezing *r*. The photon number ratios $\frac{| \beta {| }^{2}}{{\sinh }^{2}(r)}$ are plotted in Fig. 5(b).

In other words, above the level of displacement indicated by the black (or red) crosses in Fig. 5, the Taylor approximation method can efficiently estimate ${\rm{lHaf}}\left(\frac{1}{{w}^{2}}\tilde{{\boldsymbol{X}}},{{\bf{1}}}_{N}\right)$ for at least 1/2 (or 3/4) of the matrices $\tilde{{\boldsymbol{X}}}\in {\tilde{{\mathcal{G}}}}_{N\le K,K}(0)$. This reflects a transition in the average-case complexity of estimating the outcome probabilities of Uniform D-GBS, away from #P-hard, which would invalidate the proofs of hardness we present in later sections. Fig. 5 therefore, maps out a displacement regime which experiments should avoid if the goal is to demonstrate quantum advantage.

Unlike the well-defined conditions in Theorems 1 and 2, the efficient approximability of ${\rm{lHaf}}\left(\frac{1}{{w}^{2}}\tilde{{\boldsymbol{X}}},{{\bf{1}}}_{N}\right)$ in this section is conditioned on a gradual change of algorithmic success probability over matrices $\tilde{{\boldsymbol{X}}}\in {\tilde{{\mathcal{G}}}}_{N\le K,K}(0)$. It may be possible to build this into an efficient but probabilistic algorithm for simulating Uniform D-GBS, such as by the chain rule method proposed in ref. ^40^. But further techniques need to be developed to efficiently estimate the sampling probability of outcomes with photon number $N > \sqrt{M}$, as these cannot be straightforwardly expressed in terms of the $\tilde{{\boldsymbol{X}}}$ matrix. One possible method is by expanding the larger loop-Hafnians in terms of smaller ones—the loop-Hafnian of any *N* × *N* matrix can be expanded as a sum of *N* loop-Hafnians on submatrices of dimensions (*N* − 1) × (*N* − 1).

On the other hand, below the levels of displacement shown in Fig. 5, the increasing probability of success of the Taylor approximation method over matrices $\tilde{{\boldsymbol{X}}}\in {\tilde{{\mathcal{G}}}}_{N\le K,K}(0)$ does not directly translate into a proportionate algorithmic speedup in simulating instances of D-GBS. Given a Uniform D-GBS problem, due to the randomness of the matrix distribution, one cannot speed up an existing classical sampling algorithm by simply assigning the ‘easy’ instances to the efficient algorithm. In fact, if the average-case complexity of the loop-Hafnian is #P-hard, then the corresponding sampling problem plausibly remains an asymptotically hard problem, as we discuss in later sections.

One assumption we make is that the complexity transition only depends on the magnitude of ∣*w*∣. An open question is whether the complexity transition depends on the complex phase of *w* as well, which is determined by the relative phase between the displacement and squeezing parameters.

### Non-negative loop-Hafnians

A second special case we consider is ${\rm{lHaf}}(\tilde{{\boldsymbol{A}}},{\bf{1}})$ when the elements of $\tilde{{\boldsymbol{A}}}$ are non-negative. This is a special structure that has been previously exploited by classical algorithms for the efficient approximation of permanents in Fock-state Boson Sampling and Hafnians in regular GBS^41–43^.

Ref. ^25^ gives a method that approximates matching polynomials on non-negative weighted graphs based on the same Taylor approximation method. Based on this, we have the following result:

#### Theorem 3

For an *N* × *N* complex symmetric matrix $\tilde{{\boldsymbol{A}}}$ that satisfies ${\tilde{A}}_{ij}\ge 0\ \forall i,j\in [1,N],$ given any *ϵ* > 0, the function ${\rm{lHaf}}(\tilde{{\boldsymbol{A}}},{{\bf{1}}}_{N})$ can be approximated to within multiplicative error *ϵ* in time ${N}^{O(\ln N+| | \tilde{{\boldsymbol{A}}}| | -\ln \epsilon )}$.

We define the norm of $\tilde{{\boldsymbol{A}}}$ to be the maximum absolute row sum of the matrix:

19 $$| | \tilde{{\boldsymbol{A}}}| | =\mathop{\max }\limits_{i}\sum _{j\ne i}| {\tilde{A}}_{ij}| .$$

The runtime complexity ${N}^{O(\ln N+| | \tilde{{\boldsymbol{A}}}| | -\ln \epsilon )}$ grows quasi-polynomially if $| | \tilde{{\boldsymbol{A}}}| |$ grows only logarithmically. However, if $| | \tilde{{\boldsymbol{A}}}| |$ grows faster than polynomially, the runtime complexity will grow exponentially, which would render the algorithm inefficient.

Theorem 3 is particularly relevant in the search for practical applications of D-GBS. Regular GBS without displacement has been found to solve certain graph problems, such as dense-subgraph search^44–46^ and molecular docking^47,48^. These graph problems all involve a search for subgraphs with high connectivity and weights, which are preferentially sampled by GBS. Addition of loops may then be used to encode self-interaction in the graph problems, or to boost the sampling probability in a certain subregion by assigning loops to only a subset of vertices. Thus far, these application proposals have been limited to non-negative weighted graphs and as such show little advantage over classical algorithms^43^. Similarly, Theorem 3 rules out exponential quantum advantage for solving non-negative weighted graph problems by D-GBS.

We note that the runtime complexity in Theorem 3, while asymptotically efficient, can still be very large for a finite-size problem. If the ability of a quantum device that runs D-GBS to solve graph problems is compared to the performance of the Taylor approximation algorithm, the quantum device may still demonstrate a sub-exponential advantage.

### Quantum advantage via uniform D-GBS

In addition to studying where D-GBS becomes classically tractable, we also try to answer the question of where D-GBS remains classically intractable, i.e. where no efficient classical algorithm exists to sample from the same distribution. A trivial answer to this question is that D-GBS is classically intractable when it has zero displacement—the loop-Hafnian is worst-case #P-hard to estimate, and the worst case occurs when it has a zero diagonal and reduces to the Hafnian.

By connecting the loop-Hafnian to the matching polynomial in Eq. (6), however, we can already provide a better answer. Since multiplicative-error approximation of a matching polynomial is worst-case #P-hard^24,26^, by Eq. (6), so must also be a loop-Hafnian with non-zero diagonal. One can ‘hide’ the worst-case instance in the outcomes of D-GBS, since one can encode any arbitrary symmetric matrix into the ${\tilde{{\boldsymbol{B}}}}_{{\boldsymbol{n}}}$ matrices of Eq. (3). Using Stockmeyer’s theorem, one can then prove that no efficient classical algorithm can solve the D-GBS problem *exactly*, unless the Polynomial Hierarchy collapses to the third level, which is widely believed to be implausible in complexity theory^8,11,49–51^.

However, this argument is not appealing for the purpose of proving quantum advantage, as the noisy quantum devices today are themselves prevented from achieving exact precision. A more common precision target in quantum sampling schemes is defined in terms of the total-variation distance (TVD). For a target distribution ${\tilde{p}}_{{\boldsymbol{n}}}$, consider an efficient classical algorithm ${\mathcal{C}}$ that, if given some error bound *ε*, can sample from some *q*_***n***_ such that

20 $${\rm{TVD}}({\tilde{p}}_{{\boldsymbol{n}}},{q}_{{\boldsymbol{n}}})=\frac{1}{2}\mathop{\sum}_{{\boldsymbol{n}}\in \Omega }\left\vert {\tilde{p}}_{{\boldsymbol{n}}}-{q}_{{\boldsymbol{n}}}\right\vert \le \varepsilon ,$$

where Ω denotes the sample space. This additive-error approximation becomes multiplicative-error if the ${\tilde{p}}_{{\boldsymbol{n}}}$ distribution satisfies an *anti-concentration* property over ***n*** in the form of

21 $$\mathop{\Pr }\limits_{{\boldsymbol{n}}\in \Omega }\left({\tilde{p}}_{{\boldsymbol{n}}}\ge \frac{1}{\alpha | \Omega | }\right) > 1-\eta$$

for some *α*, *η* > 0. Furthermore, if the probability function ${\tilde{p}}_{{\boldsymbol{n}}}$ is *on average* #P-hard to approximate to within multiplicative error $\epsilon =O\left(\frac{\varepsilon \alpha }{\delta }\right)$ for at least a (1 − *δ*)-fraction of outcomes ***n*** ∈ Ω, then the approximate sampler ${\mathcal{C}}$ would also collapse the Polynomial Hierarchy^8,50^.

For the complexity proof of D-GBS, we utilise the Uniform D-GBS framework, which puts both the anti-concentration and average-case complexity of ${\tilde{p}}_{{\boldsymbol{n}}}$ in context of the Gaussian matrix distribution ${\boldsymbol{X}}\in {{\mathcal{G}}}_{N,K}(0,1)$ by Eqs. (12) and (13). Interestingly, we observe a transition of both properties with the amount of displacement.

The relevant probability distribution ${\tilde{p}}_{{\boldsymbol{n}}}$ we consider is that of outcomes ***n*** post-selected on $N=K\le \sqrt{M}$ photons. The post-selected probability of measuring ***n*** is derived to be SI

22 $$\begin{array}{l}{\tilde{p}}_{{\boldsymbol{n}}}=\frac{{2}^{N}}{{F}_{N}(w)}{\left| {\rm{lHaf}}\left({{\boldsymbol{U}}}_{{\boldsymbol{n}},{{\bf{1}}}_{K}}{{\boldsymbol{U}}}_{{\boldsymbol{n}},{{\bf{1}}}_{K}}^{T},w{{\boldsymbol{U}}}_{{\boldsymbol{n}},{{\bf{1}}}_{K}}{{\bf{1}}}_{N}\right)\right| }^{2},\end{array}$$

where $\frac{{2}^{N}}{{F}_{N}(w)}$ is the normalisation factor and the submatrix ${{\boldsymbol{U}}}_{{\boldsymbol{n}},{{\bf{1}}}_{K}}$ is conjectured to hide a Gaussian distribution according to Eq. (13). The function *F*_*N*_(*w*) is the convolution of absolute squared Hermite polynomial over ***n***:

23 $${F}_{N}(w)=\mathop{\sum }\limits_{{\boldsymbol{m}}\ge 0 \atop {\sum m_j=N}}\mathop{\prod}\limits_{j = 1}^{N}\frac{1}{{m}_{j}!}{\left|{H}_{{m}_{j}}\left(\frac{iw}{\sqrt{2}}\right)\right|}^{2}.$$

Since the average total photon number of Uniform D-GBS is designed to be $\overline{N}=K$, the post-selection of *N* = *K* photon-coincidence outcomes succeeds with at least inverse polynomial probability (see Supplemental MaterialsSI). Therefore, any classical algorithm that can approximately sample from the non-post-selected distribution, *p*_***n***_, can also approximately sample from ${\tilde{p}}_{{\boldsymbol{n}}}$ within polynomial time. But the latter implies the unlikely collapse of the Polynomial Hierarchy, if the loop-Hafnian in Eq. (22) is average-case #P-hard and adequately anti-concentrated. A definite proof of either property has remained elusive for various Boson Sampling proposals so far. In what follows, we make two conjectures on how these two properties for the loop-Hafnian changes with the *w* parameter from Eq. (11).

#### Conjecture 4

(Average-case complexity of the loop-Hafnian) There is some positive real $\tilde{w} > 0$, such that for all complex *w* that satisfies $| w| \le \widetilde{w}$, given any constants *ϵ*, *δ* > 0, it is #P-hard to estimate ${\rm{lHaf}}\left({{\boldsymbol{XX}}}^{T},w{\boldsymbol{X}}{{\bf{1}}}_{N}\right)$ to within multiplicative error *ϵ* with probability at least 1 − *δ* over the choice of ${\boldsymbol{X}}\in {{\mathcal{G}}}_{N,N}(0,1)$.

#### Conjecture 5

(Anti-concentration of the loop-Hafnian) There is some positive real $\tilde{w} > 0$, such that for all complex *w* that satisfies $| w| \le \widetilde{w}$, for all positive integers *N* and real *η* > 0, there exists a polynomial *α* = poly(*N*, 1/*η*) such that

24 $$\mathop{\Pr }\limits_{{\boldsymbol{X}}}\left[{\left\vert {\rm{lHaf}}\left({{\boldsymbol{XX}}}^{T},w{\boldsymbol{X}}{{\bf{1}}}_{N}\right)\right\vert }^{2} < \frac{1}{\alpha }\frac{N!{F}_{N}(w)}{{2}^{N}}\right] < \eta$$

over $${\boldsymbol{X}}\in {{\mathcal{G}}}_{N,N}(0,1)$$.

We consider the evidence for Conjectures 4 and 5, in particular noting that for smaller values of $\tilde{w}$, they are more likely to be true. More detailed discussion is provided in Methods, with further support given in Supplemental MaterialsSI.

Essentially, Conjecture 4 suggests that for some *w*, the worst-case and average-case complexity of the loop-Hafnian are equivalent and both #P-hard over ${\boldsymbol{X}}\in {{\mathcal{G}}}_{N,N}(0,1)$. Proof of average-case complexity conjectures akin to Conjecture 4 is a well-known open problem across all quantum random sampling schemes^8,11,27,50,52,53^. Established worst-to-average-case reduction techniques based on polynomial extrapolation^8,54^ do not easily extend to complex fields and have failed to converge to sufficient precision for robust quantum advantage^8,50,52^. In the Supplemental MaterialsSI, we describe how these methods can be adapted to prove exact computation of the loop-Hafnian is average-case #P-hard over finite fields, and multiplicative-error estimation is average-case #P-hard over some distributions in real fields. However, significant gaps still remain in the full proof of Conjecture 4.

Despite the lack of rigorous proof, we still believe Conjecture 4 is plausibly true for some $\tilde{w}$ value. One reason is the apparent lack of structure in the Gaussian distribution, ${\boldsymbol{X}}\in {{\mathcal{G}}}_{N,N}(0,1)$, that could enable a classical algorithm to solve a random instance more efficiently than the worst-case instance. In similar contexts, average-case and worst-case complexity of Per(***X***) (or Haf(***XX***^*T*^)) were also conjectured to be equivalent in Boson Sampling (or Gaussian Boson Sampling)^8,11^.

A second reason is the reduction of ${\rm{lHaf}}\left({{\boldsymbol{XX}}}^{T},w{\boldsymbol{X}}{{\bf{1}}}_{N}\right)$ to the Hafnian in GBS when *w* approaches 0, whose average-case approximate complexity is similarly conjectured and supported by complexity-theoretic^11^ and algorithmic evidence^12,29^.

Extending the Hafnian to the loop-Hafnian, the most obvious structure that can be exploited to separate their complexities are the diagonal weights, which is precisely what is considered previously in this work. We developed an efficient Taylor approximation method to estimate diagonally-dominant loop-Hafnians by exploiting the distribution of their zeroes. Then, in Fig. 5, we observed a transition in the algorithm’s average success probability over ${\boldsymbol{X}}\in {{\mathcal{G}}}_{N,N}(0,1)$ as *w* decreases.

We conjecture that this observation is not isolated to the specific algorithm, but that the average-case complexity of the loop-Hafnian is linked with its distribution of zeroes: the loop-Hafnian is on average easy to estimate when *w* lies in domains in the complex plane where the loop-Hafnian is on average non-zero, and #P-hard to estimate in domains where on average it contains zeroes. This is motivated by a similar transition in the matching polynomial over non-negative matrices. The zeroes of $g(z;\tilde{{\boldsymbol{A}}})$ for some non-negative real matrix $\tilde{{\boldsymbol{A}}}$ are distributed along the negative-real axis. The result is that multiplicative-error approximation to $g(z;\tilde{{\boldsymbol{A}}})$ is efficient everywhere else on the complex plane, except on the negative-real axis where the problem is #P-hard for all negative real *z* that is upper bounded by a value related to the degree of the graph^23,25,26^.

The numerical evidence based on existing methods may then suggest a possible bound of $\widetilde{w}=1$ for Conjecture 4. For at least a fraction $\delta =\frac{1}{2}$ of $\tilde{{\boldsymbol{X}}}$, when ∣*w*∣ ≤ 1, the polynomial $g(z=\frac{1}{{w}^{2}};\tilde{{\boldsymbol{X}}})$ would contain at least one zero. Thus estimation of the loop-Hafnian escapes the applicability of the Taylor approximation method. At *w* = 1, the displacement to squeezing ratio is calculated to be $\frac{| \beta {| }^{2}}{{\sinh }^{2}(r)}=5.9$, at which the loop-Hafnian also escapes the applicability of the *k*-th order approximation method proposed in ref. ^21^.

For Conjecture 5, the physical interpretation of Eq. (24) is that a non-negligible fraction of outcomes should occur with probability larger than that of a uniform distribution, and this fraction does not decay super-polynomially with *N*. This property is absent in the limiting case of ∣*w*∣ = + *∞*, which is when squeezing is reduced to zero and the input only consists of coherent states. In this limit, the probability distribution ${\tilde{{\boldsymbol{p}}}}_{{\boldsymbol{n}}}$ is given by ${\left\vert {\rm{lHaf}}\left({\bf{0}},{\boldsymbol{X}}{{\bf{1}}}_{N}\right)\right\vert }^{2}=\mathop{\prod }\nolimits_{i = 1}^{N}{\left\vert \mathop{\sum }\nolimits_{j = 1}^{K}{X}_{ij}\right\vert }^{2}$ for i.i.d. Gaussian random variables *X*_*ij*_, whose magnitudes squared follow the exponential distribution. This results in a counter-example to anti-concentration, where only an exponentially small fraction of outcomes occurring with higher than uniform probability.

When *w* is finite, the proof or disproof of Conjecture 5 becomes more difficult. When ∣*w*∣ is small, we expect the loop-Hafnian distribution to be at least as anti-concentrated as the Hafnian. In the Supplemental Materials, we present numerical evidence to support this up to ∣*w*∣ = 0.4, which corresponds to having equal mean photon number from squeezing and displacement: $\frac{| \beta {| }^{2}}{{\sinh }^{2}(r)}=1$SI, though the conjecture for higher but finite ∣*w*∣ values cannot be ruled out.

## Discussion

In this work, we have studied the change of computational complexity when a D-GBS problem becomes increasingly displaced. Exact solution by an efficient classical algorithm is not possible, because estimating the loop-Hafnian is worst-case #P-hard. With some displacement, we argue that there is a regime where even additive-precision sampling is not possible for any efficient classical algorithm, when the parameter *w*, which characterises the relative importance of displacement to that of squeezing, is small.

As ∣*w*∣ increases, we observe a transition in the complexity of D-GBS. Beyond ∣*w*∣ = 0.4, that is, as the ratio between the average photon number from displacement and from squeezing exceeds $| \beta {| }^{2}/{\sinh }^{2}(r)=1$, we note that the outcomes become increasingly concentrated in a small subset, which may enable classical spoofing strategies. Beyond ∣*w*∣ = 1 or $| \beta {| }^{2}/{\sinh }^{2}(r)=5.9$, we observe a transition in the average-case complexity of the loop-Hafnian as well. In this regime, a Taylor approximation algorithm has increasing success in estimating the sampling probability of a random outcome, until it can do so for majority of the outcomes. This invalidates the proof of hardness based on Polynomial Hierarchy collapse, and the algorithm could be used to construct an efficient classical sampler. The quoted ∣*w*∣ values are numerical estimates based on existing methods, subject to change if tighter bounds are found on the transition in Conjectures 4 and 5. The complexity transition could also depend on other factors such as mode number and number of squeezers. Eventually, when the displacement is sufficiently high such that the condition in Theorem 1 or 2 is satisfied, all outcome probabilities can be approximated to arbitrary multiplicative-error in quasi-polynomial time.

An efficient approximate solution is also possible when the D-GBS problem encodes a non-negative graph. This constrains the types of graph problems that a D-GBS scheme can solve while maintaining an exponential quantum advantage.

One important hurdle in photonic quantum computing is overcoming loss. Tensor network methods exploiting photon loss have had great success in simulating large-scale GBS demonstrations^28^. In tensor network methods, displacement is a local operation and would not change the runtime complexity. Therefore, by adding displacement without reducing their current levels of loss, experimental efforts on GBS will neither increase nor decrease the runtime separation between quantum and classical methods.

However, if loss levels can be sufficiently reduced below the simulability threshold, tensor network methods may lose out to classical algorithms that simulate the D-GBS process by loop-Hafnian calculations^12,40^. The algorithm runtime of the latter scale exponentially with the number of photons. As long as optimal classical algorithms still rely on loop-Hafnian calculations, D-GBS does present a scheme where the quantum-classical separation is increased by a factor exponential in the number of photons added by displacement.

It is also possible to simulate photon loss in GBS schemes by displacement. To sample from a mixed Gaussian state is equivalent to sampling from a pure Gaussian state with smaller mean photon number but with a random displacement^28,40^. It is therefore an interesting question for future work whether our framework of D-GBS can be used to study the complexity of lossy, un-displaced GBS. In the Supplemental Materials, we consider the link between the amount of loss required and the *w* parameter which indicates the displacement/squeezing ratio and therefore the suitability of the Taylor approximation method to simulating lossy GBS experiments SI.

Viewing displacement by a coherent state as a classical resource, our work shows that a quantum device can tolerate a certain amount of classicality without destroying its computational advantage. More importantly, since coherent states are readily generated by lasers, displacement presents a relatively easy way of scaling up the photon number—a current bottleneck in GBS experiments.

Beyond the ongoing goal of demonstrating quantum advantage, extending GBS to include displacement may support progress towards *practical* quantum advantage— solving classically intractable problems with real-world relevance. Displacement enhances the expressivity of graph-based GBS applications^19,44–48^ by enabling the encoding of loops, which models self-interaction at graph vertices. Our results suggest that such applications must go beyond non-negative graphs for exponential quantum advantage. Displacement also enables chemistry applications such as molecular vibronic spectra simulation^14–18^. While the quantum advantage of this specific problem is under active research^55^, extending the Gaussian operations to include displacement unlocks a larger space to explore for complexity-theoretically grounded quantum advantage in quantum optics.

## Methods

### Photon statistics of D-GBS

The ***B*** matrix and ***γ*** vector in Eq. (1) are given by refs. ^10,22^:

25 $$\left(\begin{array}{ll}{\boldsymbol{B}}&0\\ 0&{{\boldsymbol{B}}}^{* }\end{array}\right)=\left(\begin{array}{ll}0&{{\mathbb{1}}}_{M}\\ {{\mathbb{1}}}_{M}&0\end{array}\right)\left({{\mathbb{1}}}_{2M}-{{\boldsymbol{\Sigma }}}_{Q}^{-1}\right),$$

26 $$\left(\begin{array}{ll}{\gamma }^{T}&{\gamma }^{\dagger }\end{array}\right)={{\boldsymbol{\mu }}}^{\dagger }{{\boldsymbol{\Sigma }}}_{Q}^{-1}.$$

For an *M*-mode pure state constructed by applying an interferometer transformation, ***U*** on displaced squeezed states, $| \psi \left.\right\rangle =\hat{U}{({\boldsymbol{U}})\bigoplus }_{j = 1}^{M}{\hat{D}}_{j}({\beta }_{j}){\hat{S}}_{j}({r}_{j})| 0\left.\right\rangle$, the pair (***B***, ***γ***) is given by refs. ^10,22^ (detailed derivations also presented in the Supplemental MaterialsSI):

27 $${\boldsymbol{B}}={{\boldsymbol{U}}\,\bigoplus }_{j = 1}^{M}\tanh ({r}_{j})\,{{\boldsymbol{U}}}^{T},$$

28 $${\boldsymbol{\gamma }}={\boldsymbol{U}}{{\boldsymbol{\beta }}}^{* }-{\boldsymbol{U}}\left({\bigoplus }_{j = 1}^{M}\tan h({r}_{j})\right){\boldsymbol{\beta }}.$$

In a general D-GBS problem, the matrix $\tilde{{\boldsymbol{B}}}$, defined in Eq. (4) for easier mapping to the matching polynomial, is the ***B*** matrix rescaled by the an outer product of the ***γ*** vector with itself. By Eqs. (27) and (28), we see that the magnitude $\left\vert {\tilde{B}}_{ij}\right\vert$ increases (decreases) monotonically with ∣*r*_*j*_∣ (∣*β*_*j*_∣) and thus represents a ratio of squeezing to displacement.

This ratio is further simplified to the *w* parameter in Eq. (11) by Eqs. (8) and (9) in the Uniform D-GBS model. It is straightforward to derive Eqs. (8) and (9) from Eqs. (27) and (28), if we make use of the following requirements:

29 $${r}_{j}=\left\{\begin{array}{ll}r,\quad{\rm{for}}\,1\le j\le K,\\ 0,\quad{\rm{for}}\,j > K;\end{array}\right.$$

30 $${\beta }_{j}=\left\{\begin{array}{ll}\beta ,\quad{\rm{for}}\,1\le j\le K,\\ 0,\quad{\rm{for}}\,j > K.\end{array}\right.$$

The photon number distribution of a single-mode displaced squeezed state, $| {\psi }_{j}\left.\right\rangle ={\hat{D}}_{j}(\beta ){\hat{S}}_{j}(r)| 0\left.\right\rangle$, is given by refs. ^56,57^:

31 $$\begin{array}{ll}{p}^{(j)}(n)={{\rm{e}}}^{-| \beta {| }^{2}+\Re ({\beta }^{2})\tanh (r)}\frac{{\tanh }^{n}(r)}{n!{2}^{n}\cosh (r)} {\left| {H}_{n}\left(\frac{{\beta }^{* }\cosh (r)-\beta \sinh (r)}{-i\sqrt{\sinh (2r)}}\right)\right| }^{2},\end{array}$$

where *H*_*n*_ is the *n*-th Hermite polynomial in the physicist’s definition and the variable inside the Hermite polynomial can be rewritten in terms of the *w* parameter from Eq. (11). The probability of measuring *N* photons across *K* identically displaced squeezed states is then given by a discrete convolution over the *K* modes, with vacuum probability given by

32 $${p}_{{\bf{0}}}=\frac{1}{{\cosh }^{K}(r)}{{\rm{e}}}^{-K| \beta {| }^{2}+K\tanh (r)\Re ({\beta }^{2})}$$

which derives the post-selected probability, ${\tilde{p}}_{{\boldsymbol{n}}}$, in Equation (22).

### Taylor approximation to the loop-Hafnian

Here, we give an overview of the Taylor approximation method used in the main text for estimating diagonally dominant and non-negative loop-Hafnians, with a derivation of its runtime complexity. Detailed explanation can also be found in refs. ^25,30^. The scheme works for any general polynomial of degree *d*:

33 $$g(z)={a}_{0}+{a}_{1}z+\ldots +{a}_{d}{z}^{d}.$$

The matching polynomial, $g(z;\tilde{{\boldsymbol{A}}})={\rm{lHaf}}(z\tilde{{\boldsymbol{A}}},{{\bf{1}}}_{N})$, on an *N* × *N* matrix $\tilde{{\boldsymbol{A}}}$, has degree $d=\lfloor \frac{N}{2}\rfloor$.

If the roots of *g*(*z*) are ${\zeta }_{1},\ldots ,{\zeta }_{d}\in {\mathbb{C}}$, then *g*(*z*) can be rewritten as

34 $$g(z)={a}_{d}(z-{\zeta }_{1})\ldots (z-{\zeta }_{d}).$$

If there is a disc in complex plane with radius *R* where the polynomial is non-zero, i.e. ∣*ζ*_*i*_∣ > *R* for all 1 ≤ *i* ≤ *d*, then we can define a function *f* to be the logarithm of *g*(*z*):

35 $$f(z)=\ln g(z)=\ln ({a}_{d})+\ln (z-{\zeta }_{1})+\ldots +\ln (z-{\zeta }_{d}).$$

Function *f* can be Taylor expanded as

36 $$f(z)=\mathop{\sum }\limits_{j=1}^{\infty }\frac{{z}^{j}}{j!}{f}^{(j)}(0)=f(0)-\mathop{\sum }\limits_{j=1}^{\infty }\frac{{t}_{j}{z}^{j}}{j},$$

where $${t}_{j}=\mathop{\sum }\nolimits_{i = 1}^{d}{\zeta }_{i}^{-j}$$.

If we truncate the Taylor expansion at degree *m*:

37 $${T}_{m}(z)=f(0)-\mathop{\sum }\limits_{j=1}^{m}\frac{{t}_{j}{z}^{j}}{j},$$

then we can approximate *f*(*z*) to additive error:

38 $$| f(z)-{T}_{m}(z)| =\left| \mathop{\sum}\limits_{j=m+1}^{\infty }\frac{{t}_{j}{z}^{j}}{j}\right|t \le \frac{1}{m+1}\mathop{\sum }\limits_{j=m+1}^{\infty }| {t}_{j}{z}_{j}| .$$

Since the roots of the polynomial lie outside radius *R* in complex plane, ∣*ζ*_*j*_∣ > *R*, ∀ *j* ∈ [1, *d*], the absolute value of the *j*-th inverse power sum is bounded by $| {t}_{j}| =\left\vert \mathop{\sum }\nolimits_{i = 1}^{d}{\zeta }_{i}^{-j}\right\vert \le d/{R}^{j}$. The additive error is therefore bounded by the sum of a geometric progression,

39 $$| f(z)-{T}_{m}(z)| =\frac{d}{m+1}\frac{{q}^{m+1}}{1-q},$$

where the ratio *q* is given by *q* = ∣*z*∣/*R* < 1.

Given any error bound *ϵ* > 0, we can choose $m=C(\ln d-\ln \epsilon )$, where the constant *C* can be chosen as *C* = (1−*q*)^−1^. Then

40 $$\frac{d}{m+1}\frac{{q}^{m+1}}{1-q} < d{q}^{m}=d{\left(\frac{d}{\epsilon }\right)}^{C\ln q}d{\left(\frac{d}{\epsilon }\right)}^{-1}=\epsilon .$$

To derive the runtime complexity of the algorithm for the loop-Hafnian, we make use of the loop-Hafnian expansion formula derived in refs. ^10,21^. For some *N* × *N* matrix $\tilde{{\boldsymbol{A}}}$, one can express ${\rm{lHaf}}(\tilde{{\boldsymbol{A}}},{{\bf{1}}}_{N})$ as

41 $${\rm{lHaf}}(\tilde{{\boldsymbol{A}}},{{\bf{1}}}_{N})=\mathop{\sum }\limits_{m = 0}^{\lfloor N/2\rfloor }\mathop{\sum }\limits_{{S\subseteq \{1,\ldots ,N\}}\atop {| S| = 2m}}{\rm{Haf}}({\tilde{{\boldsymbol{A}}}}_{S}),$$

where *S* is a set of integer numbers with cardinality 2*m* < *N*, and ${\tilde{{\boldsymbol{A}}}}_{S}$ is a submatrix of $\tilde{{\boldsymbol{A}}}$ constructed by keeping the *i*-th row and column of $\tilde{{\boldsymbol{A}}}$ if and only if *i* ∈ *S*. If $S=\varnothing$, then ${\rm{Haf}}({\tilde{{\boldsymbol{A}}}}_{\varnothing })=1$. The matching polynomial can therefore be rewritten in a similar form,

42 $$g(z;\tilde{{\boldsymbol{A}}})=\mathop{\sum}\limits_{m = 0}^{\lfloor N/2\rfloor }\left(\mathop{\sum }\limits_{{S\subseteq \{1,\ldots ,N\}}\atop {| S| = 2m}}{\rm{Haf}}({\tilde{{\boldsymbol{A}}}}_{S})\right){z}^{m},$$

whose *m*-th order derivative is:

43 $${g}^{(m)}(0;\tilde{{\boldsymbol{A}}})=m!\mathop{\sum }\limits_{{S\subseteq \{1,\ldots ,N\}}\atop {| S| = 2m}}{\rm{Haf}}({\tilde{{\boldsymbol{A}}}}_{S}).$$

The runtime of computing *T*_*m*_(*z*) scales with the coefficient of its leading term, $\frac{{d}^{m}}{d{z}^{m}}\ln g(0)$, which can be computed in polynomial number of calls to Eq. (43)^25^. Since Eq. (43) can be computed by direct enumeration in time ${N}^{O(m)}={N}^{O(\ln N-\ln \epsilon )}$, the function *T*_*m*_(*z*) can also be computed in quasi-polynomial time ${N}^{O(\ln N-\ln \epsilon )}$.

### Complexity proof of uniform D-GBS

Consider the classical algorithm ${\mathcal{C}}$ with precision that satisfies Eq. (20). Then, by Stockmeyer’s theorem, one can compute some ${\tilde{q}}_{{\boldsymbol{n}}}$ that approximates *q*_***n***_ to a multiplicative error, e.g. $\left\vert {\tilde{q}}_{{\boldsymbol{n}}}-{q}_{{\boldsymbol{n}}}\right\vert \le 2\varepsilon {q}_{{\boldsymbol{n}}}$, in BPP^NP^^8,49^. By using Markov’s inequality,

44 $$\mathop{\Pr }\limits_{{\boldsymbol{n}}\in \Omega }\left(\left\vert {q}_{{\boldsymbol{n}}}-{\tilde{p}}_{{\boldsymbol{n}}}\right\vert \ge \frac{4\varepsilon }{k| \Omega | }\right)\le \frac{k}{2},$$

45 $$\mathop{\Pr }\limits_{{\boldsymbol{n}}\in \Omega }\left({q}_{{\boldsymbol{n}}}\ge \frac{2}{k| \Omega | }\right)\le \frac{k}{2},$$

one derives that ${\tilde{q}}_{{\boldsymbol{n}}}$ is also an approximation to ${\tilde{p}}_{{\boldsymbol{n}}}$, but to an additive error,

46 $$\left\vert {\tilde{q}}_{{\boldsymbol{n}}}-{\tilde{p}}_{{\boldsymbol{n}}}\right\vert \le \left\vert {\tilde{q}}_{{\boldsymbol{n}}}-{q}_{{\boldsymbol{n}}}| +| {q}_{{\boldsymbol{n}}}-{\tilde{p}}_{{\boldsymbol{n}}}\right\vert \le \frac{8\varepsilon }{k}\frac{1}{| \Omega | },$$

with probability at least 1 − *k* over ***n*** ∈ Ω.

Equation (46) becomes a multiplicative-error approximation to ${\tilde{p}}_{{\boldsymbol{n}}}$, if the target distribution ${\tilde{p}}_{{\boldsymbol{n}}}$ further satisfies an anti-concentration property over ***n*** in the form of Eq. (21). In this case, Eq. (46) can be rewritten as

47 $$\left\vert {\tilde{q}}_{{\boldsymbol{n}}}-{\tilde{p}}_{{\boldsymbol{n}}}\right\vert \le \frac{8\varepsilon \alpha }{k}{\tilde{p}}_{{\boldsymbol{n}}}$$

with probability at least 1 − *k* − *η* over ***n*** ∈ Ω. If the probability function ${\tilde{p}}_{{\boldsymbol{n}}}$ is *on average* #P-hard to approximate to within multiplicative error $\epsilon =O\left(\frac{\varepsilon \alpha }{\delta }\right)$ for at least a (1 − *δ*)-fraction of outcomes ***n*** ∈ Ω, then the approximate sampler ${\mathcal{C}}$ would collapse the Polynomial Hierarchy to the third level.

In some complexity proofs for quantum random sampling schemes, the constants *ϵ* and *δ* in Conjecture 4 are given precise values^27^, while we keep them in their generic form. To some extent, they can be traded off against each other and against the $\widetilde{w}$ value. More importantly, they can also be traded off against *ε* and *α* in Eq. (47), which defines the precision of the adversarial classical algorithm and how anti-concentrated ${\tilde{p}}_{{\boldsymbol{n}}}$ needs to be to rule it out. If the loop-Hafnian is average-case #P-hard for polynomial multiplicative error, *ϵ* = poly(*N*), then classical samplers with even polynomial TVD precision, *ε* = poly(*N*), are ruled out, and the *α* in Eq. (21) can be polynomial in *N*. In the Supplemental Materials, we provide a proof that multiplicative-error approximation of the loop-Hafnian is indeed *worst-case* #P-hard for polynomial error bounds, *ϵ*≤poly(*N*) SI. But we leave the question open on whether this could be conjectured for the average-case as well.

A similar trade-off exists for the anti-concentration conjecture. If the loop-Hafnian lacks anti-concentration entirely, e.g. if $\alpha =\Omega (\exp (N))$ in Eq. (24), then the adversarial classical algorithm needs to be exponentially precise in TVD $\varepsilon =\frac{1}{\exp (N)}$ (Eq. (47)), in order to collapse the Polynomial Hierarchy. In other words, the quantum experiment needs to be exponentially close to the ground-truth distribution to guarantee an escape of classical simulability. On the other hand, if a stronger version of Conjecture 5 can hold true for some D-GBS variant, e.g. if *α* is constant in Eq. (24), then any adversarial classical sampler with constant TVD precision could be ruled out by assuming the Polynomial Hierarchy doesn’t collapse.

A recent study on the Hafnian has also revealed a loss of anti-concentration in GBS if the number of initially-squeezed modes, *K*, scales too slowly with total mode number, *M*^58^. A similar transition could also exist in D-GBS, but if the anti-concentration *K* scale more quickly than $K=\sqrt{M}$, the output distribution may no longer ‘hide’ a Gaussian distribution of matrices. The average-case complexity of the loop-Hafnian needs then be studied over a different distribution of matrices. However, for a sufficiently random distribution, we still expect equivalence between the worst-case and the average-case complexity of the loop-Hafnian when displacement is small.

### Towards experimental implementations

Displacement of a single mode can be experimentally implemented by interfering the single-mode quantum state with a strong local oscillator on a highly asymmetric beamsplitter^59,60^. The proposed Uniform D-GBS scheme requires identical displacement operations on all non-vacuum input modes, so that we can separate the *w* parameter from the Gaussian submatrices of the Haar random unitary. These displacement operations can be implemented by an extra *M* beamsplitters and phase-locked coherent states with individual control over their reflectivities.

Identical displacement on all populated input modes is an assumption made for mathematical convenience and is not a necessary condition for the D-GBS problem to be hard. If this requirement is dropped, ref. ^21^ showed that a single-mode coherent state is sufficient to apply displacements to a multi-mode state provided both are inputted into a fully connected interferometer, in which every input mode is able to interfere with every other input mode. Alternatively, displacement with a single coherent state may also be achieved by seeding the non-linear process that generates the squeezed vacuum state with a coherent state in the target wavelength^61,62^. In either case, the action of the interferometer applies displacements to each mode depending on its transfer matrix, which in general can be non-zero for every mode. This then enables the same rewriting of the loop-Hafnian as the matching polynomial, albeit on a different distribution of matrices from the Uniform D-GBS scheme.

In a similar vein, the requirement for the number of non-vacuum input modes to be upper bounded by $K\le \sqrt{M}$ was also made for mathematical convenience and to target a Gaussian distribution in the loop-Hafnian matrices. Increasing *K* beyond this bound could be experimentally beneficial when the squeezing operations are marred by photon loss^28^. Provided the new distribution is sufficiently random and is absent of special structures, we expect a similar reduction of the average-case to worst-case complexity of estimating the loop-Hafnian.

In our paper, we discussed two special matrix structures that would allow efficient estimation of the loop-Hafnian: diagonal-dominance and non-negativity. Another example is the sparsity. An interferometer with only limited connectivity, instead of the full connectivity that quantum advantage would stipulate, reduces the loop-Hafnian calculations to sparser matrices that allow for more efficient classical algorithms^13,63,64^.

Applying displacement via the interferometer couples the covariance matrix ***Σ*** and the mean vector ***μ*** defining the output state. To generate arbitrary displacement independent of the interferometer, one needs to revert back to the use of *M* additional individually controlled beamsplitters. The ability of individually controlling squeezing, displacement and interferometer enables encoding of any arbitrary complex symmetric matrix into the D-GBS scheme. Therefore, if there is a specific distribution of matrices, on which stronger evidence is found for average-case complexity and the anti-concentration of the loop-Hafnian, one can *design* a quantum advantage experiment with D-GBS from this distribution.

Our complexity proof required the total average photon number to be upper bounded by square root of the number of modes, $\overline{N}\le \sqrt{M}$, such that the photon statistics remain collision-less. If this requirement were relaxed, collisions would introduce repetitions to the rows and columns of fd(***B***_***n***_, ***γ***_***n***_), which increases the dimensions of the matrix without increasing its rank. However, with a moderate amount of collisions, there is no evidence that the sampling problem becomes efficient to solve. The collision-less condition is also required in regular GBS proposals^11^, although recent work suggests it may not be required for the hardness of Fock-basis Boson Sampling^65^. We leave as open question for future work the implications of relaxing the collision-less requirement for D-GBS.

## Supplementary information

Supplementary Information

## Data Availability

The numerical data underlying the results presented in this paper are available from the corresponding author on reasonable request.
