# Supplementary material for: A complexity transition in displaced Gaussian Boson sampling
Source: npj Quantum Inf. 2025 Jul 9;11(1):119. doi: 10.1038/s41534-025-01062-5 (PMC12240864; doi:10.1038/s41534-025-01062-5)
Supplement: Supplementary file 1 — Supplementary Information [file 41534_2025_1062_MOESM1_ESM.pdf]

Supplemental Materials: A Complexity Transition in Displaced Gaussian Boson Sampling

Zhenghao Li,[1,*] Naomi R. Solomons,[2,3,†] Jacob F.F. Bulmer,[3] Raj B. Patel,[1] and Ian A. Walmsley[1]

[1] *Department of Physics, Imperial College London, Prince Consort Road, London SW7 2AZ, United Kingdom*

[2] *Quantum Engineering Centre for Doctoral Training, Centre for Nanoscience and Quantum Information, University of Bristol, Bristol, BS8 1FD, United Kingdom*

[3] *Quantum Engineering Technology Labs, H. H. Wills Physics Laboratory and Department of Electrical and Electronic Engineering, University of Bristol, Bristol BS8 1UB, United Kingdom*

CONTENTS

SI. Photon statistics of GBS 1
- A. Photon statistics of a Gaussian state 1
- B. GBS matrices 2
- C. Photon statistics of Uniform D-GBS 3
- D. Post-selection probability for complexity proof 4

SII. Approximation schemes for the loop-Hafnian 5
- A. Proof of Theorem 1 5
- B. Proof of Theorem 2 6

SIII. Complexity of the loop-Hafnian 7
- A. Worst-case complexity 7
- B. Average-case complexity 8

SIV. Loop-Hafnian Anti-Concentration 10

SV. Modelling a noisy channel as random displacements 13

References 16

SI. PHOTON STATISTICS OF GBS

A. Photon statistics of a Gaussian state

We perform calculations in the Fock basis, where the basis vector is $\hat{\boldsymbol{\xi}} = \begin{pmatrix}\hat{a}_1 & \ldots \hat{a}_M & \hat{a}_1^\dagger & \ldots & \hat{a}_M^\dagger\end{pmatrix}^T$ and satisfies the bosonic commutation relations $[\hat{\boldsymbol{\xi}}, \hat{\boldsymbol{\xi}}^\dagger] = \begin{pmatrix} 0_M & \mathbb{1}_M \\ -\mathbb{1}_M & 0 \end{pmatrix}$, where $\mathbb{1}_M$ is an $M \times M$ identity matrix. An M-mode Gaussian state of light, ρ, can be fully described by a $2M \times 2M$ covariance matrix $\boldsymbol{\Sigma}$ and a length-$2M$ means vector $\boldsymbol{\mu}$ [1]:

$$\mu_\nu = \langle \hat{\xi}_\nu \rangle, \tag{S1a}$$

$$\Sigma_{\nu\tau} = \frac{1}{2}\langle \{\hat{\xi}_\nu, \hat{\xi}_\tau^\dagger\}\rangle - \langle \hat{\xi}_\nu \rangle \langle \hat{\xi}_\tau^\dagger \rangle, \tag{S1b}$$

where the subscripts ν and τ run from 1 to $2M$.

---

* zhenghao.li21@imperial.ac.uk

† Present address: LIP6, CNRS, Sorbonne Université, 4 Place Jussieu, F-75005 Paris, France

The probability of projecting the Gaussian state ρ onto an M-mode Fock state $|\boldsymbol{n}\rangle = |n_1, \ldots, n_M\rangle$ is given by[2, 3]:

$$p_{\boldsymbol{n}} = \langle \boldsymbol{n}| \rho |\boldsymbol{n}\rangle = \frac{p_{\boldsymbol{0}}}{\prod_{j=1}^{M} n_j!} \times \mathrm{lHaf}\left(\boldsymbol{A}_{\boldsymbol{n}}, \boldsymbol{\Gamma}_{\boldsymbol{n}}\right). \tag{S2}$$

The vacuum probability, $p_{\boldsymbol{0}}$, is given by

$$p_{\boldsymbol{0}} = \frac{\exp\left(-\frac{1}{2}\boldsymbol{\mu}^\dagger \boldsymbol{\Sigma}_Q^{-1}\boldsymbol{\mu}\right)}{\sqrt{\det(\boldsymbol{\Sigma}_Q)}}, \tag{S3}$$

where $\boldsymbol{\Sigma}_Q = \boldsymbol{\Sigma} + \frac{\mathbb{1}}{2}$ is the Husimi covariance matrix. The $\boldsymbol{A}$ matrix and $\boldsymbol{\Gamma}$ vector are defined as

$$\boldsymbol{A} = \begin{pmatrix} 0 & \mathbb{1}_M \\ \mathbb{1}_M & 0 \end{pmatrix} \left(\mathbb{1}_{2M} - \boldsymbol{\Sigma}_Q^{-1}\right), \tag{S4a}$$

$$\boldsymbol{\Gamma}^T = \boldsymbol{\mu}^\dagger \boldsymbol{\Sigma}_Q^{-1}. \tag{S4b}$$

The vector $\boldsymbol{\Gamma}$ has the form $\boldsymbol{\Gamma} = \begin{pmatrix} \gamma \\ \gamma^* \end{pmatrix}$. If the Gaussian state is undisplaced, then $\boldsymbol{\mu} = \boldsymbol{0}$ and hence $\boldsymbol{\Gamma} = \boldsymbol{0}$.

For a pure Gaussian state, the $\boldsymbol{A}$ matrix has a block diagonal form, $\boldsymbol{A} = \begin{pmatrix} \boldsymbol{B} & 0 \\ 0 & \boldsymbol{B}^* \end{pmatrix}$ [2]. Then the probability of measuring $\boldsymbol{n}$ becomes

$$p_{\boldsymbol{n}} = \frac{p_{\boldsymbol{0}}}{\prod_{j=1}^{M} n_j!} \times \left|\mathrm{lHaf}\left(\boldsymbol{B}_{\boldsymbol{n}}, \boldsymbol{\gamma}_{\boldsymbol{n}}\right)\right|^2. \tag{S5}$$

B. GBS matrices

We use the following definitions for the squeezing and displacement operators:

$$\hat{S}(r) = \exp\left(\frac{r}{2}(\hat{a}^{\dagger 2} - \hat{a}^2)\right), \tag{S6}$$

$$\hat{D}(\beta) = \exp\left(\beta \hat{a}^\dagger - \beta^* \hat{a}\right), \tag{S7}$$

where we have assumed the squeezing parameter is real. We also define the actions of the interferometer to be

$$\hat{U}^\dagger(\boldsymbol{U})\hat{\boldsymbol{\xi}}\hat{U}(\boldsymbol{U}) = \begin{pmatrix} \boldsymbol{U}^* & 0 \\ 0 & \boldsymbol{U} \end{pmatrix} \hat{\boldsymbol{\xi}}. \tag{S8}$$

The covariance matrix and means vector for the M-mode pure state, $|\psi\rangle = \hat{U}(\boldsymbol{U}) \bigoplus_{j=1}^{M} \hat{D}_j(\beta_j)\hat{S}_j(r_j)\,|0\rangle$, is given by

$$\boldsymbol{\mu} = \begin{pmatrix} \boldsymbol{U}^* & 0 \\ 0 & \boldsymbol{U} \end{pmatrix} \begin{pmatrix} \boldsymbol{\beta} \\ \boldsymbol{\beta}^* \end{pmatrix} = \begin{pmatrix} \boldsymbol{U}^*\boldsymbol{\beta} \\ \boldsymbol{U}\boldsymbol{\beta}^* \end{pmatrix}, \tag{S9a}$$

$$\boldsymbol{\Sigma} = \tfrac{1}{2}\begin{pmatrix} \boldsymbol{U}^* & 0 \\ 0 & \boldsymbol{U} \end{pmatrix} \begin{pmatrix} \bigoplus \cosh(2r_j) & \bigoplus \sinh(2r_j) \\ \bigoplus \sinh(2r_j) & \bigoplus \cosh(2r_j) \end{pmatrix} \begin{pmatrix} \boldsymbol{U}^T & 0 \\ 0 & \boldsymbol{U}^\dagger \end{pmatrix} \tag{S9b}$$

$$= \tfrac{1}{2}\begin{pmatrix} \boldsymbol{U}^* & 0 \\ 0 & \boldsymbol{U} \end{pmatrix} \boldsymbol{L} \begin{pmatrix} \bigoplus \mathrm{e}^{2r_j} & 0 \\ 0 & \bigoplus \mathrm{e}^{-2r_j} \end{pmatrix} \boldsymbol{L}^\dagger \begin{pmatrix} \boldsymbol{U}^T & 0 \\ 0 & \boldsymbol{U}^\dagger \end{pmatrix}, \tag{S9c}$$

where $\boldsymbol{\beta} = \left(\beta_1, \ldots, \beta_M\right)^T$ and $\bigoplus$ is short for $\bigoplus_{j=1}^{M}$. The matrix $\boldsymbol{L}$ is a unitary matrix $\boldsymbol{L} = \frac{1}{\sqrt{2}}\begin{pmatrix} \mathbb{1}_M & i\mathbb{1}_M \\ \mathbb{1}_M & -i\mathbb{1}_M \end{pmatrix}$.

The determinant of the Husimi covariance matrix $\boldsymbol{\Sigma}_Q = \boldsymbol{\Sigma} + \frac{\mathbb{1}}{2}$ is then easy to calculate:

$$\det(\boldsymbol{\Sigma}_Q) = \prod_{j=1}^{M}\left(\frac{1}{2} + \frac{1}{2}\mathrm{e}^{2r_j}\right)\left(\frac{1}{2} + \frac{1}{2}\mathrm{e}^{-2r_j}\right) = \prod_{j=1}^{M}\cosh(r_j)^2. \tag{S10}$$

The inverse Husimi covariance matrix is

$$\boldsymbol{\Sigma}_Q^{-1} = \begin{pmatrix} \mathbb{1}_M & -\boldsymbol{U}^* \bigoplus \tanh(r_j)\boldsymbol{U}^\dagger \\ -\boldsymbol{U} \bigoplus \tanh(r_j)\boldsymbol{U}^T & \mathbb{1}_M \end{pmatrix}, \tag{S11}$$

which allows us to find the expressions for $(\boldsymbol{A}, \boldsymbol{\Gamma})$:

$$\boldsymbol{A} = \begin{pmatrix} \boldsymbol{U} \bigoplus \tanh(r_j)\boldsymbol{U}^T & 0 \\ 0 & \boldsymbol{U}^* \bigoplus \tanh(r_j)\boldsymbol{U}^\dagger \end{pmatrix}, \tag{S12a}$$

$$\boldsymbol{\Gamma} = \begin{pmatrix} \boldsymbol{U}\boldsymbol{\beta}^* - \boldsymbol{U}\left(\bigoplus \tanh(r_j)\right)\boldsymbol{\beta} \\ \boldsymbol{U}^*\boldsymbol{\beta} - \boldsymbol{U}^*\left(\bigoplus \tanh(r_j)\right)\boldsymbol{\beta}^* \end{pmatrix}, \tag{S12b}$$

or the pure state pair $(\boldsymbol{B}, \boldsymbol{\gamma})$:

$$\boldsymbol{B} = \boldsymbol{U} \bigoplus_{j=1}^{M} \tanh(r_j)\boldsymbol{U}^T, \tag{S13a}$$

$$\boldsymbol{\gamma} = \boldsymbol{U}\boldsymbol{\beta}^* - \boldsymbol{U}\left(\bigoplus \tanh(r_j)\right)\boldsymbol{\beta}. \tag{S13b}$$

C. Photon statistics of Uniform D-GBS

Following the Uniform D-GBS scheme proposed in the main text, and using Equations S3, S9a, S10 and S11, we can derive the vacuum probability to be

$$p_{\mathbf{0}} = \frac{\exp\left(-K|\beta|^2 + K\tanh(r)\Re(\beta^2)\right)}{\cosh(r)^K}, \tag{S14}$$

where the symbol $\Re(\cdot)$ denotes taking the real part.

The photon number distribution of a single-mode displaced squeezed state $|\psi_j\rangle = \hat{D}_j(\beta)\hat{S}_j(r)|0\rangle$ is given by [4, 5]

$$p^{(j)}(n) = \exp\left(-|\beta|^2 + \Re(\beta^2)\tanh(r)\right)\frac{\tanh^n(r)}{n!2^n\cosh(r)} \times \left|H_n\left(\frac{\beta^*\cosh(r) - \beta\sinh(r)}{-i\sqrt{\sinh(2r)}}\right)\right|^2, \tag{S15}$$

where superscript (j) denotes the mode number. H_n is the n-th Hermite polynomial in the physicist's definition, given by

$$H_n(x) = (-1)^n \mathrm{e}^{x^2}\frac{d^n}{dx^n}\mathrm{e}^{-x^2}. \tag{S16}$$

The variable inside the Hermite polynomial can be rewritten in terms of the w parameter, $w = \frac{\beta^* - \beta\tanh(r)}{\sqrt{\tanh(r)}}$, defined in the main text:

$$\frac{\beta^*\cosh(r) - \beta\sinh(r)}{-i\sqrt{\sinh(2r)}} = \frac{iw}{\sqrt{2}}. \tag{S17}$$

The probability of measuring N photons across K identically displaced squeezed states is then given by a discrete convolution over the K modes:

$$p_N = \sum_{\substack{\boldsymbol{m}\geq 0 \\ \sum m_i = N}} \prod_{j=1}^{K} p^{(j)}(m_j) = p_{\mathbf{0}}\tanh^N(r)\frac{1}{2^N}F_{N,K}(w). \tag{S18}$$

The sum $\sum_{\substack{\boldsymbol{m}\geq 0 \\ \sum m_i=N}}$ is over all length-K vectors $\boldsymbol{m}=(m_1,\ldots,m_K)$ such that $m_i \geq 0$ for all $i\in[1,K]$ and $\sum_{i=1}^K m_i = N$. The big F function is given by

$$F_{N,K}(w) = \sum_{\substack{\boldsymbol{m}\geq 0 \\ \sum m_i=N}} \prod_{j=1}^{K} \frac{1}{m_j!} \left| H_{m_j}\left(\frac{iw}{\sqrt{2}}\right)\right|^2 . \tag{S19}$$

And if $N=K$, we abbreviate the notation to $F_N(w)$, which has its definition given in the main text.

One can also check that if we set $w=0$, Equation S18 gives us the photon number distribution for K identical single-mode squeezed vacuum states [2, 6]:

$$p_N = \binom{\frac{N}{2}+\frac{K}{2}-1}{\frac{N}{2}} \operatorname{sech}^K(r)\tanh^N(r) \text{ for } N \text{ is even.} \tag{S20}$$

D. Post-selection probability for complexity proof

In the main text, we proved that if Conjectures 4 and 5 are true, then Uniform D-GBS cannot be approximately simulated by an efficient classical sampler unless the Polynomial Hierarchy collapses to the third level. For mathematical convenience, we post-selected on the $N=K=\overline{N}$ photon-coincidence outcomes, where $\overline{N}$ is the total mean photon number. If the post-selection succeeds with at least inverse polynomial probability, then simulating the complete, non-post-selected distribution is at least as hard as simulating the post-selected distribution. In this supplemental section, we show explicitly that this post-selection succeeds with at least inverse polynomial probability.

First, we consider the displacement component. The photon-number distribution from K coherent states follow a Poissonian distribution with mean $\overline{N}_{\text{dis}}$ [7]:

$$p^{(\text{dis})}(N) = \frac{(\overline{N}_{\text{dis}})^N}{N!} \mathrm{e}^{-\overline{N}_{\text{dis}}}. \tag{S21}$$

Using Stirling's approximation [8], $N! \sim \sqrt{2\pi N}\left(\frac{N}{e}\right)^N$ for large N, we find that $p^{(\text{dis})}(\overline{N}_{\text{dis}})$ scales as $1/\sqrt{2\pi\overline{N}_{\text{dis}}}$, assuming $\overline{N}_{\text{dis}}$ is an integer.

Next, we consider the squeezing component. In Uniform D-GBS model, this consists of K identical single-mode squeezed vacuum states, with photon number distribution given by Equation S20. The mean photon number from the squeezed states is $\overline{N}_{\text{sq}} = K\sinh^2(r)$. For mathematical simplicity, let's assume $\overline{N}_{\text{sq}} = 2\nu$ and $K=2\mu$ for two integers ν,μ. Then Equation S20 for $N=\overline{N}_{\text{sq}}$ can be rewritten as:

$$p^{(\text{sq})}\left(\overline{N}_{\text{sq}}\right) = \binom{\nu+\mu-1}{\nu}\left(\frac{\mu}{\nu+\mu}\right)^{\mu}\left(\frac{\nu}{\nu+\mu}\right)^{\nu} \tag{S22}$$

Using Stirling's approximation for large ν and μ, the binomial coefficient scales as $\binom{\nu+\mu-1}{\nu} \sim \sqrt{\frac{\nu+\mu-1}{2\pi\nu(\mu-1)}} \times \frac{(\nu+\mu-1)^{\nu+\mu-1}}{\nu^\nu(\mu-1)^{\mu-1}}$. Combining this with Equation S22, we find that $p^{(\text{sq})}\left(\overline{N}_{\text{sq}}\right)$ scales inverse polynomially with $\overline{N}_{\text{sq}}$, assuming $\overline{N}_{\text{sq}}$ is an even integer.

Combining the displacement and squeezing components in Uniform D-GBS, the total mean photon number is the sum of that from each component: $\overline{N} = \overline{N}_{\text{dis}} + \overline{N}_{\text{sq}}$. The post-selection probability on $N=\overline{N}$ is lower bounded by $p_{\overline{N}} \geq p^{(\text{dis})}(\overline{N}_{\text{dis}}) \times p^{(\text{sq})}(\overline{N}_{\text{sq}})$. A fixed w parameter, defined in Equation 11 in the main text, defines a linear relationship between $\overline{N}_{\text{dis}}$ (or $\overline{N}_{\text{sq}}$) and $\overline{N}$. Therefore, the post-selection probability must scale at least inverse polynomially with $\overline{N}$.

The above analysis assumed that the mean photon numbers, $\overline{N}, \overline{N}_{\text{dis}}$, and $\overline{N}_{\text{sq}}$, are (even) integers. But the scaling would not change qualitatively if we relax these assumptions and round to the nearest (even) integer photon number.

The post-selection is only a tool for mathematical convenience in the complexity proof. We can also post-select on all photons $N \leq K = \overline{N}$, for which Conjecture 4 then needs to be reworded as average-case #P-hardness over matrices $\boldsymbol{X} \in \mathcal{G}_{N,K}(0,1)$ for $N \leq K$. This is the matrix distribution, over which the Hafnian was conjectured to be average-case #P-hard to estimate in the complexity proof for regular GBS [6].

SII. APPROXIMATION SCHEMES FOR THE LOOP-HAFNIAN

As explained in the main text, in order to prove that the loop-Hafnian is efficient to approximate in special cases, one only needs to prove that the special case falls inside a non-negative region in the complex plane, which enables approximation by the Taylor approximation algorithm. The next two subsections prove Theorem 1 and Theorem 2 by this method.

A. Proof of Theorem 1

Theorem 1 and its proof is given in Ref. [9] for any partition function based on an abstract polymer model. Here, we give a summary of the theory and adapt it to the loop-Hafnian.

In an abstract polymer model, we consider a finite set of polymers $\mathcal{K}$. Let $\sim$ be a compatibility relation. A pair of compatible polymers are denoted by $\gamma_1 \sim \gamma_2$. A polymer is always incompatible with itself: $\gamma \not\sim \gamma$. Let $\mathcal{D}$ be the set of all subsets $\Phi \subseteq \mathcal{K}$ consisting of mutually compatible polymers. Let's also denote a weight function $w : \mathcal{K} \to \mathbb{C}$ and define a partition function as

$$Z(w) = \sum_{\Phi \in \mathcal{D}} \prod_{\gamma \in \Phi} w(\gamma). \tag{S23}$$

Ref. [9] proved the following theorem:

Theorem S1. *Let two functions $a, d : \mathcal{K} \to \mathbb{R}^+$, and weight function $w : \mathcal{K} \to \mathbb{C}$ be such that*

$$\sum_{\gamma' : \gamma' \not\sim \gamma} |w(\gamma')| \, \mathrm{e}^{a(\gamma') + d(\gamma')} \leq a(\gamma) \tag{S24}$$

for any $\gamma \in \mathcal{K}$, then $Z(w) \neq 0$

We can adapt the abstract polymer model to the loop Hafnian, $\mathrm{lHaf}(\tilde{\boldsymbol{A}}, \mathbf{1}_N)$. The matrix $\tilde{\boldsymbol{A}}$ defines the adjacency matrix of a loopless graph $G(V, E)$. The finite set of polymers is the set of edges E. Each edge, $e \in E$ is a polymer and two edges, e_1, e_2, are compatible if they *do not* share a common vertex, $e_1 \cap e_2 = \emptyset$. Then $\mathcal{D}$ is the set of all matchings. The weight function for an edge, $e = (i, j)$, is its edge weight, $w(e) = \tilde{A}_{ij}$, and the partition function $Z(w)$ in Equation S23 is the loop Hafnian $\mathrm{lHaf}(\tilde{\boldsymbol{A}}, \mathbf{1}_N)$.

We choose two constant functions such that for every edge, $e \in E$, $a(e) = a$ and $d(e) = d$ for $a, d \in \mathbb{R}^+$. Then we have

$$\sum_{e_2 : e_2 \cap e_1 \neq \emptyset} |w(e_2)| \mathrm{e}^{a(e_2) + d(e_2)} \leq (2N - 3) \mathrm{e}^{a+d} \max(|\tilde{A}_{ij}|), \tag{S25}$$

where $2N - 3$ is the maximum number of edges that share a common vertex with edge e_1 and hence the number of incompatible edges with e_1. In order to satisfy the inequality in Theorem S1, we require

$$\max(|\tilde{A}_{ij}|) \leq \frac{a}{\mathrm{e}^{a+d}(2N - 3)}. \tag{S26}$$

And when $\max(|\tilde{A}_{ij}|) \leq \frac{1}{\mathrm{e}(2N-3)}$, we can always find suitable $a, d \in \mathbb{R}^+$ to satisfy this inequality. We therefore have the following lemma.

Lemma S2. *Given an $N \times N$ complex symmetric matrix $\tilde{\boldsymbol{A}}$ whose matrix elements satisfy*

$$|\tilde{A}_{ij}| \leq \frac{1}{\mathrm{e}(2N - 3)} \; \forall \, i \neq j \tag{S27}$$

Then $\mathrm{lHaf}(\tilde{\boldsymbol{A}}, \mathbf{1}_N) \neq 0$.

We are now ready to prove Theorem 1 in the main text.

Proof. For some positive real $0 < \lambda < 1$, if $|A_{ij}| < \frac{\lambda}{\mathrm{e}(2N-3)} \, \forall i \neq j$, then the matching polynomial $g(z; \tilde{\boldsymbol{A}}) \neq 0$ for $|z| < \frac{1}{\lambda}$ by Lemma S2. This defines a disc in complex plane with radius $R = \frac{1}{\lambda}$ that includes $z = 1$. Thus, by the Taylor approximation algorithm, $g(z = 1; \tilde{\boldsymbol{A}})$ can be approximated to arbitrary multiplicative error by truncating the Taylor expansion of its logarithm. This truncated series can be calculated in time $N^{O_\lambda(\ln N - \ln \epsilon)}$ time, where the scaling in O_λ only depends on λ. □

B. Proof of Theorem 2

We use a lemma given and proved in Ref. [10]:

Lemma S3. *Let us fix $\alpha_1, \ldots, \alpha_N \in \mathbb{C}$. Then for any $a_1, \ldots, a_N \in \mathbb{C}$ there exist $a'_1, \ldots, a'_N \in \mathbb{C}$ such that*

$$\sum_{k=1}^{N} \alpha_k a_k = \sum_{k=1}^{N} \alpha_k a'_k, \; \sum_{k=1}^{N} |a'_k| \leq \sum_{k=1}^{N} |a_k| \tag{S28}$$

and $a'_k \neq 0$ for at most one k.

In a similar fashion as the proof for Theorem 1, we first prove the following lemma

Lemma S4. *Given an $N \times N$ complex symmetric matrix $\tilde{\boldsymbol{A}}$ that satisfies*

$$\sum_{j \neq i} |\tilde{A}_{ij}| < \frac{1}{N-1} \; \forall i \in [1, N]. \tag{S29}$$

Then $\mathrm{lHaf}(\tilde{\boldsymbol{A}}, \mathbf{1}_N) \neq 0$.

The proof for Lemma S4 is inspired by Ref. [10] for diagonally dominant permanents.

Proof. Let Θ_N be the set of complex symmetric $N \times N$ matrices $\tilde{\boldsymbol{A}}$ satisfying $\sum_{j \neq i} |\tilde{A}_{ij}| < \frac{1}{N-1} \; \forall i \in [1, N]$. We claim for every $\tilde{\boldsymbol{A}} \in \Theta_N$, there exists $\tilde{\boldsymbol{A}}' \in \Theta_N$, such that $\mathrm{lHaf}(\tilde{\boldsymbol{A}}, \mathbf{1}_N) = \mathrm{lHaf}(\tilde{\boldsymbol{A}}', \mathbf{1}_N)$, and $\tilde{\boldsymbol{A}}'$ has at most one non-zero off-diagonal entry in row j *and* column j for all $j = 1, \ldots, N$. In other words, in a graph whose adjacency matrix is given by $\mathrm{fd}(\tilde{\boldsymbol{A}}', \mathbf{1}_N)$, every vertex can at most be connected to itself and one other vertex.

This $\tilde{\boldsymbol{A}}'$ can be constructed by modifying each row and column of $\tilde{\boldsymbol{A}}$. Let $\tilde{\boldsymbol{A}}_{-\{i,\ldots,k\}}$ denote the submatrix of $\tilde{\boldsymbol{A}}$, where the $i, \ldots, k$-th row and column are removed. In the first step, we expand $\mathrm{lHaf}(\tilde{\boldsymbol{A}}, \mathbf{1}_N)$ by its first row and column:

$$\mathrm{lHaf}(\tilde{\boldsymbol{A}}, \mathbf{1}_N) = 1 \times \mathrm{lHaf}(\tilde{\boldsymbol{A}}_{-\{1\}}, \mathbf{1}_{N-1}) + \sum_{j=2}^{N} \tilde{A}_{1j} \, \mathrm{lHaf}(\tilde{\boldsymbol{A}}_{-\{1,j\}}, \mathbf{1}_{N-2}). \tag{S30}$$

Applying Lemma S3, there exists $\tilde{A}^{(1)}_{1j}$ for $j = 2, \ldots, N$, such that

$$\sum_{j=2}^{N} \tilde{A}_{1j} \, \mathrm{lHaf}(\tilde{\boldsymbol{A}}_{-\{1,j\}}, \mathbf{1}_{N-2}) = \sum_{j=2}^{N} \tilde{A}^{(1)}_{1j} \, \mathrm{lHaf}(\tilde{\boldsymbol{A}}_{-\{1,j\}}, \mathbf{1}_{N-2}), \tag{S31}$$

where

$$\sum_{j=2}^{N} |\tilde{A}^{(1)}_{1j}| \leq \sum_{j=2}^{N} |\tilde{A}_{1j}| < \frac{1}{N-1}, \tag{S32}$$

and $\tilde{A}^{(1)}_{1j} \neq 0$ for at most one value of $j \neq 1$. We replace the elements of the first row and column of $\boldsymbol{A}$ with $\tilde{A}^{(1)}_{1j}$. The new matrix is denoted as $\tilde{\boldsymbol{A}}^{(1)}$, which satisfies

$$\mathrm{lHaf}(\tilde{\boldsymbol{A}}, \mathbf{1}_N) = \mathrm{lHaf}(\tilde{\boldsymbol{A}}^{(1)}, \mathbf{1}_N). \tag{S33}$$

In the k-th step, for $2 \leq k \leq N-1$, we expand $\mathrm{lHaf}\, \tilde{\boldsymbol{A}}^{(k-1)}, \mathbf{1}_N)$ by the k-th row:

$$\mathrm{lHaf}(\tilde{\boldsymbol{A}}^{(k-1)}, \mathbf{1}_N) = 1 \times \mathrm{lHaf}(\tilde{\boldsymbol{A}}^{(k-1)}_{-\{k\}}, \mathbf{1}_{N-1}) + \sum_{i<k} \tilde{A}^{(i)}_{ki} \, \mathrm{lHaf}(\tilde{\boldsymbol{A}}^{(k-1)}_{-\{k,i\}}, \mathbf{1}_{N-2}) + \sum_{i>k} \tilde{A}_{ki} \, \mathrm{lHaf}(\tilde{\boldsymbol{A}}^{(k-1)}_{-\{k,i\}}, \mathbf{1}_{N-2}), \tag{S34}$$

where elements $\tilde{A}^{(i)}_{ki}$ have been replaced in the i-th step for $i < k$. Applying Lemma S3, there exists $\tilde{A}^{(k)}_{kj}$ for $j \neq k$, such that

$$\sum_{i<k} \tilde{A}^{(i)}_{ki} \, \mathrm{lHaf}(\tilde{\boldsymbol{A}}^{(k-1)}_{-\{k,i\}}, \mathbf{1}_{N-2}) + \sum_{i>k} \tilde{A}_{ki} \, \mathrm{lHaf}(\tilde{\boldsymbol{A}}^{(k-1)}_{-\{k,i\}}, \mathbf{1}_{N-2}) = \sum_{j \neq k} \tilde{A}^{(k)}_{kj} \, \mathrm{lHaf}(\tilde{\boldsymbol{A}}^{(k-1)}_{-\{k,j\}}, \mathbf{1}_{N-2}), \tag{S35}$$

where

$$\sum_{j\neq k}^{N} |\tilde{A}_{kj}^{(k)}| \leq \sum_{i<k} |\tilde{A}_{ki}^{(i)}| + \sum_{i>k} |\tilde{A}_{ki}| < \frac{k}{N-1}, \tag{S36}$$

and $\tilde{A}_{kj}^{(k)} \neq 0$ for at most one value of $j \neq k$. The upper bound $\frac{k}{N-1}$ in Equation S36 is the worst case bound, taken when $|\tilde{A}_{ki}^{(i)}| \neq 0$ for all $i < k$, each of which upper bounded by $\frac{1}{N-1}$. And because $\sum_{i>k} |\tilde{A}_{ki}| < \frac{1}{N-1}$, the total upper bound is $\frac{k}{N-1}$. When $k = N$, however, the term $\sum_{i>k} |\tilde{A}_{ki}|$ doesn't exist, so the upper bound in Equation S36 becomes $\frac{N-1}{N-1} = 1$. In the best case, where $|\tilde{A}_{ki}^{(i)}| = 0$ for all $i < k$, then Equation S36 is upper bounded by only $\frac{1}{N-1}$. At the end of this step, we replace the k-th row and column of $\tilde{\boldsymbol{A}}^{(k-1)}$ with $\tilde{A}_{kj}^{(k)}$. The new matrix is denoted as $\tilde{\boldsymbol{A}}^{(k)}$.

At the end of the k-th step, the i-th row and column of the matrix $\tilde{\boldsymbol{A}}^{(k)}$, for all $i < k$, cannot have more than one non-zero entry. If $\tilde{A}_{ki}^{(i)} = 0$ before the k-th step, then it could be taken out of the summation in Equation S35. If $\tilde{A}_{ki}^{(i)} \neq 0$ before the k-th step, then it is possible to have $\tilde{A}_{ki}^{(k)} = \tilde{A}_{ik}^{(k)} \neq 0$ after the k-th step, but all the other elements along the i-th row and column will be 0 by the i-th step of the procedure.

If $i > k$, then it is possible for the i-th row and column of $\tilde{\boldsymbol{A}}^{(k)}$ to have more than one non-zero entries. However, they will be overwritten in the later i-th step.

At the end of the N-th step, where all N rows and columns have been overwritten, we have constructed a new matrix $\tilde{\boldsymbol{A}}^{(N)}$ such that

$$\mathrm{lHaf}(\tilde{\boldsymbol{A}}^{(N)}, \mathbf{1}_N) = \mathrm{lHaf}(\tilde{\boldsymbol{A}}, \mathbf{1}_N). \tag{S37}$$

Each row and column in $\tilde{\boldsymbol{A}}^{(N)}$ will have at most *one* non-zero off-diagonal entry and that entry is bounded by Equation S36. Thus, if we denote the off-diagonal elements of $\tilde{\boldsymbol{A}}^{(N)}$ as $(\tilde{\boldsymbol{A}}^{(N)})_{ij}$, we have

$$\sum_{j\neq i} \left|(\tilde{\boldsymbol{A}}^{(N)})_{ij}\right| < 1 \;\forall\; i \in [1, N]. \tag{S38}$$

Consider the graph defined by $\mathrm{fd}(\tilde{\boldsymbol{A}}^{(N)}, \mathbf{1}_N)$. Every vertex in the graph is connected to itself via a loop of weight 1, and is connected to at most one another vertex via an edge of weight bounded by Equation S38. In other words, the graph is a set of disjoint subgraphs $G_1, \ldots, G_{N'}$, where each subgraph G_j either consists of one single vertex ($|V(G_j)| = 1$) connected to itself via loop of weight 1; or consists of one pair of vertices ($|V(G_j)| = 2$), each with a loop of weight 1 and connected to each other via an edge of weight $|w(j)| < 1$. As a result, the loop Hafnian $\mathrm{lHaf}(\tilde{\boldsymbol{A}}^{(N)}, \mathbf{1}_N)$ is

$$\mathrm{lHaf}(\tilde{\boldsymbol{A}}^{(N)}, \mathbf{1}_N) = \prod_{\substack{j=1 \\ |V(G_j)|=2}}^{N'} (1 + w(j)) \neq 0, \tag{S39}$$

thus completing the proof. □

Given Lemma S4, the proof for Theorem 2 follows the same recipe as that for Theorem 1.

SIII. COMPLEXITY OF THE LOOP-HAFNIAN

A. Worst-case complexity

In the context of monomer dimer systems, the problem of counting dimer arrangements (or matchings) is known to be worst-case #P-complete [11]. The #P complexity class are counting problems associated with NP problems. It is proven that multiplicative-error approximation to the matching polynomial is worst-case #P-hard [12]. By the equivalence between the matching polynomial and the loop-Hafnian with diagonal of ones, $\mathrm{lHaf}(\tilde{\boldsymbol{A}}, \mathbf{1}_N)$, one can deduce that multiplicative-error approximation to the latter is also worst-case #P-hard.

The same result can also be proven using a technique in Ref. [13], which proved that multiplicative-error approximation of the Permanent is worst-case #P-hard. We give only a sketch of the proof here. For details of the proof, the reader is referred to Ref. [13]. The idea of the proof is if there exists an oracle $\mathcal{O}$ that, given a matrix $\tilde{\boldsymbol{A}} \in \mathbb{R}^{N\times N}$,

approximates $\mathrm{lHaf}^2(\tilde{\boldsymbol{A}}, \mathbf{1}_N)$ to within multiplicative error $\epsilon < \mathrm{poly}(N)$, then, given a matrix $\boldsymbol{C} \in \{0,1\}^{N\times N}$, one can compute $\mathrm{lHaf}(\boldsymbol{C}, \mathbf{1}_N)$ exactly in polynomial time and using $O((\epsilon+1)N^2 \ln N)$ adaptive queries to $\mathcal{O}$. But we know the exact computation of $\mathrm{lHaf}(\boldsymbol{C}, \mathbf{1}_N)$ is #P-complete by Ref. [11]. Therefore, multiplicative error approximation of $\mathrm{lHaf}^2(\tilde{\boldsymbol{A}}, \mathbf{1}_N)$ must be #P-hard.

Let $\boldsymbol{Y}$ be the bottom-right $(N-1)\times(N-1)$ submatrix of $\boldsymbol{C}$. Given a real number r, let $\mathbf{1}_N^{[r]}$ be a vector where the first element is $1-r$ and the rest are 1. Our key observation is

$$\mathrm{lHaf}(\boldsymbol{C}, \mathbf{1}_N^{[r]}) = \mathrm{lHaf}(\boldsymbol{C}, \mathbf{1}_N) - r\,\mathrm{lHaf}(\boldsymbol{Y}, \mathbf{1}_{N-1}). \tag{S40}$$

This is the reason we can use the proof technique in Ref. [13], which builds up a Permanent by the same recursion relationship. The loop-Hafnian, $\mathrm{lHaf}(\boldsymbol{C}, \mathbf{1}_N^{[r]})$, can be rewritten as a loop-Hafnian on a matrix with diagonal of ones:

$$\mathrm{lHaf}(\boldsymbol{C}, \mathbf{1}_N^{[r]}) = (1-r)\,\mathrm{lHaf}(\tilde{\boldsymbol{A}}, \mathbf{1}_N), \tag{S41}$$

where $\tilde{A}_{1j} = \tilde{A}_{j1} = \frac{C_{1j}}{1-r}$ and $\tilde{A}_{ij} = C_{ij}$ for all other elements. Therefore, the oracle $\mathcal{O}$ can be used to estimate $\mathrm{lHaf}(\boldsymbol{C}, \mathbf{1}_N^{[r]})$.

The proof builds $\mathrm{lHaf}(\boldsymbol{C}, \mathbf{1}_N)$ recursively in N steps. Inductively, we assume we already know $\mathrm{lHaf}(\boldsymbol{Y}, \mathbf{1}_{N-1})$. Then, in order to calculate $\mathrm{lHaf}(\boldsymbol{C}, \mathbf{1}_N)$, one can do so by finding a $r^* \in \mathbb{R}$ such that $\mathrm{lHaf}(\boldsymbol{C}, \mathbf{1}_N^{[r^*]}) = 0$. Once we find such r^*, we can calculate $\mathrm{lHaf}(\boldsymbol{C}, \mathbf{1}_N) = r^*\,\mathrm{lHaf}(\boldsymbol{Y}, \mathbf{1}_{N-1})$. Since $\boldsymbol{C} \in \{0,1\}^{N\times N}$ and $\boldsymbol{Y} \in \{0,1\}^{(N-1)\times(N-1)}$, the ratio r^* must be a rational number. What Ref. [13] proved is that, by a binary search procedure, the rational number r^* can be found by $O((\epsilon+1)N\ln N)$ queries to oracle $\mathcal{O}$, which is used to estimate $\mathrm{lHaf}(\boldsymbol{C}, \mathbf{1}_N^{[r]})$. Multiplied by the recursion steps, one can calculate $\mathrm{lHaf}(\boldsymbol{C}, \mathbf{1}_N)$ in $O((\epsilon+1)N^2\ln N)$ queries to $\mathcal{O}$ in total, which would complete the proof.

Given that multiplicative-approximation to the loop-Hafnian is worst-case #P-hard, by using Stockmeyer's theorem [14] and the technique in Ref. [13], one can already prove that no classical algorithm can efficiently *exactly* simulate Displaced GBS, unless the Polynomial Hierarchy collapses.

B. Average-case complexity

In Conjecture 4 in the main text, we conjectured that there is some $\tilde{w}$, such that multiplicative-error approximation to the loop-Hafnian, $\mathrm{lHaf}\left(\boldsymbol{X}\boldsymbol{X}^T, w\boldsymbol{X}\mathbf{1}_N\right)$ is average-case #P-hard for $|w| \leq \tilde{w}$. The average-case is taken over the i.i.d. Gaussian distribution of $\boldsymbol{X} \in \mathcal{G}_{N,N}(0)$ matrices. By Equation 14 in the main text, this is equivalent to the average-case approximate hardness of $\mathrm{lHaf}\left(\frac{1}{w^2}\tilde{\boldsymbol{X}}, \mathbf{1}_N\right)$ over $\tilde{\boldsymbol{X}} \in \tilde{\mathcal{G}}_{N,N}(0)$.

In Section SIII A, we proved that the loop-Hafnian is worst-case #P-hard to estimate. Significant challenges exist for proving its average-case approximate hardness. Indeed, a successful worst-to-average-case reduction for estimating the Permanent (or the Hafnian) has remained notoriously elusive for (Gaussian) Boson Sampling [6, 13, 15]. The same challenge also persists for other quantum random sampling schemes [15–17]. While a definitive proof lies outside the scope of this work, we discussed in the main text the various reasons why Conjecture 4 might be true, which we supplement in this section.

A polynomial extrapolation technique exists for the worst-to-average-case reduction for the *exact* hardness of the Permanent. Using this technique, it can be proved that the Permanent over a large *finite field*, such as a set of integers modulo a prime number, is as hard to exactly compute on average as it is in the worst case [13, 18]. This method relies on embedding the worst-case instance inside a univariate polynomial of some indeterminate t. When t is non-zero, the polynomial is the Permanent of uniform random matrices sampled from the finite field. When $t = 0$, the polynomial reduces to the Permanent of the worst-case instance. Given some oracle that can compute the Permanent over a majority of random matrices within the finite field, one can then reconstruct the univariate polynomial with polynomial number of calls to the oracle. After this, one can then extrapolate to find the polynomial value at $t = 0$, which equals the Permanent of the worst-case instance. Therefore, the worst-case and average-case complexity of computing the Permanent over the finite field must be equivalent.

A key reason this technique works is because the Permanent of a matrix with entries that are low-degree polynomials in the indeterminate x is itself also a low-degree polynomial [18]. This enables efficient reconstruction of the polynomial. The same is also true for the Hafnian and the loop-Hafnian, which is why it is straightforward to prove that *exact* computation of the loop-Hafnian is average-case #P-hard over finite fields.

Using the average-case exact hardness of the loop-Hafnian over finite fields, one can find *some* distribution $\mathcal{D}$ over real matrices $\tilde{\boldsymbol{A}} \in \mathbb{R}^{N\times N}$, such that multiplicative-error estimation of $\mathrm{lHaf}^2(\tilde{\boldsymbol{A}}, \mathbf{1}_N)$ for most matrices $\tilde{\boldsymbol{A}} \in \mathcal{D}$ is #P-hard. In Section SIII A, we showed how the computation of loop-Hafnian over matrices in a finite field can be reduced

to the multiplicative-error estimation of $\mathrm{lHaf}^2(\tilde{\boldsymbol{A}}, \mathbf{1}_N)$, for some matrices $\tilde{\boldsymbol{A}} \in \mathbb{R}^{N\times N}$. We can use this reduction technique to *define* the distribution $\mathcal{D}$ from the finite field. Thus, given any oracle that estimates $\mathrm{lHaf}^2(\tilde{\boldsymbol{A}}, \mathbf{1}_N)$ for majority of matrices $\tilde{\boldsymbol{A}} \in \mathcal{D}$, the loop-Hafnian over majority of matrices in the finite field can be exactly computed with polynomial calls to the oracle and thereby solve a #P-hard problem. However, distribution $\mathcal{D}$ will be far different from the physically-motivated construction of matrix distributions in Uniform D-GBS.

Directly adapting worst-to-average-case reduction techniques to $\mathrm{lHaf}\left(\frac{1}{w^2}\tilde{\boldsymbol{X}}, \mathbf{1}_N\right)$ for $\tilde{\boldsymbol{X}} \in \tilde{\mathcal{G}}_{N,N}(0)$ is difficult, a challenge also shared by complexity proofs for the Permanent [13, 15, 19]. First, distribution $\tilde{\mathcal{G}}_{N,N}(0)$ is now defined over the complex field, which invalidates certain proof techniques from finite field analysis. But suppose we can still construct a univariate polynomial $q(t) = \mathrm{lHaf}\left(\frac{1}{w^2}\tilde{\boldsymbol{A}}(t), \mathbf{1}_N\right)$, such that $\tilde{\boldsymbol{A}}(t) \in \tilde{\mathcal{G}}_{N,N}(0)$ is a random Gaussian matrix when $t \neq 0$, and $\tilde{\boldsymbol{A}}(0) = \tilde{\boldsymbol{A}}$ reduces to some worst-case instance $\tilde{\boldsymbol{A}}$ when $t = 0$. A second challenge is an oracle that can only *approximately* estimate $\mathrm{lHaf}\left(\frac{1}{w^2}\tilde{\boldsymbol{X}}, \mathbf{1}_N\right)$ leaves uncertainty in the reconstruction of the univariate polynomial. Yet, the polynomial extrapolation method is exponentially sensitive to uncertainty over the complex field. Average-case instances estimated with very small errors can still lead to exponential errors when extrapolated to estimate the worst-case instance [19].

A further hurdle is, in order for the polynomial extrapolation technique to work over $\tilde{\boldsymbol{X}} \in \tilde{\mathcal{G}}_{N,N}(0)$, one requires the entries to the loop-Hafnian to be approximately random matrices from $\tilde{\mathcal{G}}_{N,N}(0)$, for all instances of t. Ref. [13] showed such a polynomial can be constructed for i.i.d. Gaussian matrices. The same might also be true for $\tilde{\mathcal{G}}_{N,N}(0)$, since the distribution is defined by Gaussian matrices (Equation 15 in the main text). But even so, Ref. [13] can only prove the average-case *exact* hardness of Gaussian Permanents due to the aforementioned challenges.

The various challenges in proving average-case approximate complexity for the loop-Hafnian possibly implies the needs of seeking alternative techniques beyond the polynomial extrapolation method. This remains an interesting open question for future research on D-GBS as well as other quantum random sampling schemes.

In the absence of a rigorous average-case complexity proof, in the main text we detailed why we believe Conjecture 4 might be true for small displacements possibly up to $\tilde{w} = 1$.

One argument is that in the extreme limit of $w \to 0$, the loop-Hafnian $\mathrm{lHaf}\left(\boldsymbol{X}\boldsymbol{X}^T, w\boldsymbol{X}\mathbf{1}_N\right)$ reduces to the Hafnian [20] of the form $\mathrm{Haf}(\boldsymbol{X}\boldsymbol{X}^T)$ for Gaussian matrices $\boldsymbol{X} \in \mathcal{G}_{N,K}(0,1)$, whose modulus squared gives the probability distribution of un-displaced GBS schemes. Various complexity-theoretic, algorithmic and experimental evidence have been provided for its average-case approximate hardness [6, 21–25].

A second argument is that for classical algorithms to achieve a speed-up in estimating the loop-Hafnian over the Hafnian, they must exploit some structure that is present in the former but absent in the latter. The most obvious structure of such are the loop weights, which are reduced to the single parameter w in our model. How to exploit this parameter for algorithmic speed-up is precisely what the first half of our paper studies: when $|w|$ is very large, we showed that multiplicative-error approximation of $\mathrm{lHaf}\left(\boldsymbol{X}\boldsymbol{X}^T, w\boldsymbol{X}\mathbf{1}_N\right)$ is on average *not* #P-hard. We show this by presenting a quasi-polynomial algorithm, whose success probability gradually increases as $|w|$ increases beyond $|w| = 1$.

We conjecture that this transition is not isolated to the specific algorithm that we propose, and that the problem should have a phase where it remains average-case #P-hard for a certain range of non-zero w. From the current evidence, for $w = 1$ the problem already escapes the capability of classical methods, both of that proposed in this work and that in Ref. [26], though it is possible that better classical algorithms may further bring down this budget for displacement.

A further reason for believing estimation of $\mathrm{lHaf}\left(\frac{1}{w^2}\tilde{\boldsymbol{X}}, \mathbf{1}_N\right)$ being average-case #P-hard for small $|w|$ is because it is worst-case #P-hard, and we conjecture that the distribution of $\tilde{\boldsymbol{X}}$ does not provide extra structure to separate the worst-case and the average-case. However, even if it does and some other distribution doesn't, one could potentially encode the alternative distribution into $\tilde{\boldsymbol{B}}_{\boldsymbol{n}}$ in a different D-GBS setup.

As an example, we could require an alternative D-GBS setup to have $\gamma_i = 1$ for all i. Suitable displacement parameters can be found by solving Equation S13b. If the $\boldsymbol{B}_{\boldsymbol{n}}$ matrices still have the form of $\boldsymbol{B}_{\boldsymbol{n}} = \tanh(r)\boldsymbol{U}_{\boldsymbol{n},\mathbf{1}_K}\boldsymbol{U}_{\boldsymbol{n},\mathbf{1}_K}^T$, then the matrices $\tilde{\boldsymbol{B}}_{\boldsymbol{n}}$ now has the distribution of

$$\tilde{\boldsymbol{B}}_{\boldsymbol{n}} = \tanh(r)\boldsymbol{U}_{\boldsymbol{n},\mathbf{1}_K}\boldsymbol{U}_{\boldsymbol{n},\mathbf{1}_K}^T \sim \tanh(r)\boldsymbol{X}\boldsymbol{X}^T \tag{S42}$$

for $\boldsymbol{X} \in \mathcal{G}_{N,N}(0, 1/M)$. The probability distribution of this alternative D-GBS scheme would thus be given by

$$p_{\boldsymbol{n}} \propto \left|\mathrm{lHaf}(\tilde{\boldsymbol{B}}_{\boldsymbol{n}}, \mathbf{1}_N)\right|^2 \sim \left|\mathrm{lHaf}(\tanh(r)\boldsymbol{X}\boldsymbol{X}^T, \mathbf{1}_N)\right|^2. \tag{S43}$$

Another alternative D-GBS setup would be to keep the Uniform D-GBS design, but increase the number of non-vacuum input modes, K, to be linear with total number of modes, M. The corresponding probability distribution is

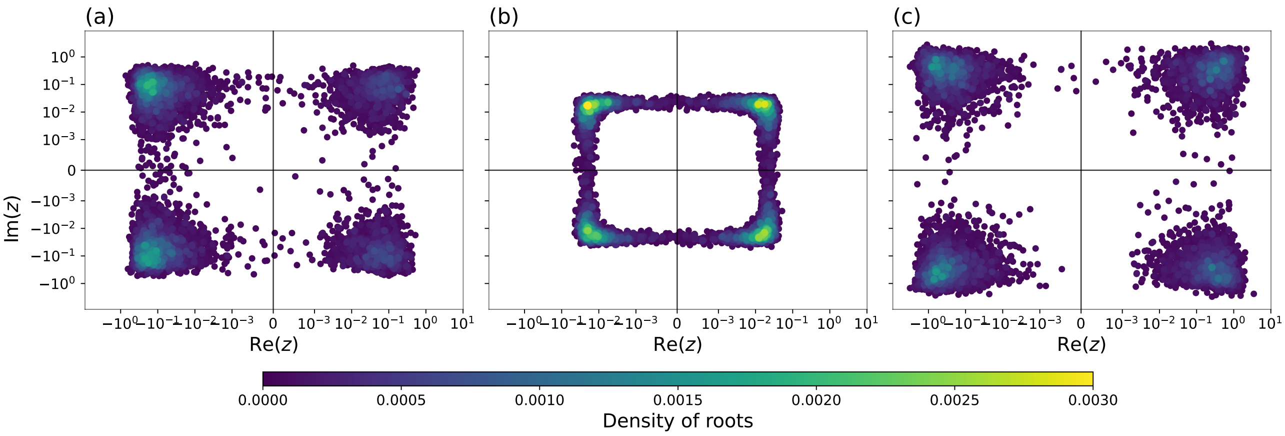

FIG. S1. Here, we plot the distribution of zeroes for $g(z;\tilde{\boldsymbol{A}}) = \mathrm{lHaf}(z\tilde{\boldsymbol{A}}, \boldsymbol{1}_N)$ in the complex plane for different distributions of $\tilde{\boldsymbol{A}}$. In each plot, the matching polyonmial is calculated for 10,000 randomly drawn $\tilde{\boldsymbol{A}}$ matrices, and the root with minimum magnitude is scatter plotted. (a) $\tilde{\boldsymbol{A}} = \tilde{\boldsymbol{X}} \in \tilde{\mathcal{G}}_{N=12,K=12}(0)$ (Uniform D-GBS from the main text); (b) $\tilde{\boldsymbol{A}} = \boldsymbol{X}\boldsymbol{X}^T$ for i.i.d. Gaussian matrices $\boldsymbol{X} \in \mathcal{G}_{N=12,K=12}(0,1)$; (c) $\tilde{\boldsymbol{A}} = \frac{\boldsymbol{U}_{\boldsymbol{n},K}\boldsymbol{U}_{\boldsymbol{n},K}^T}{(\boldsymbol{U}_{\boldsymbol{n},K}\boldsymbol{1}_N)(\boldsymbol{U}_{\boldsymbol{n},K}\boldsymbol{1}_N)}$ for $N \times K$ submatrices of random $M = M$ unitaries, where $N = 12, K = M = 144$.

still described by:

$$p_{\boldsymbol{n}} \propto \left|\mathrm{lHaf}\left(\boldsymbol{U}_{\boldsymbol{n},\boldsymbol{1}_K}\boldsymbol{U}_{\boldsymbol{n},\boldsymbol{1}_K}^T, w\boldsymbol{U}_{\boldsymbol{n},\boldsymbol{1}_K}\boldsymbol{1}_N\right)\right|^2, \tag{S44}$$

but submatrices $\boldsymbol{U}_{\boldsymbol{n},\boldsymbol{1}_K}$ no longer hide a Gaussian distribution.

The distribution of zeroes for these alternative distributions are shown in Fig. S1. There is a similar change of density of zeroes as z move away from the origin of the complex plane as that in Fig. 4 in the main text. Again this indicates an increasing success probability of the Taylor approximation method in estimating $\mathrm{lHaf}(z\tilde{\boldsymbol{A}}, \boldsymbol{1}_N)$ as $|z|$ decreases, which correspond to a decrease in the squeezing to displacement ratio in D-GBS.

Finally, we noted in the main text that un-displaced GBS schemes with photon loss can be decomposed into a mixed state D-GBS with a random displacement [27, 28]. In Section SV, we show the positive correlation between the loss factor and the median displacement. This implies the complexity equivalence of low-loss GBS and low-displacement D-GBS: given access to some oracle that estimates the loop-Hafnian over majority of instances of the random displacement distribution, one can approximately simulate lossy GBS with the corresponding loss level. However, current complexity and algorithmic evidence suggests that GBS with sufficiently low loss remains beyond classical simulability [6, 29], which can serve as a further motivation for Conjecture 4.

SIV. LOOP-HAFNIAN ANTI-CONCENTRATION

In Conjecture 5 in the main text, we conjectured that the loop-Hafnian is sufficiently anti-concentrated over the distribution of $\boldsymbol{X} \in \mathcal{G}_{N,N}(0,1)$. We want to *avoid* a scenario where a large 'concentration' of loop-Hafnian values are close to zero, and this concentration grows exponentially with N.

By way of example, let's consider a D-GBS problem with a large fraction of outcomes that occur with near-zero probability. There is only a small fraction of outcomes that occur with a non-negligible probability and this fraction shrinks exponentially with the scale of the problem, N. An adversial classical algorithm can then approximate the D-GBS problem by only sampling from the small fraction of outcomes with non-negligible probability. And even though the runtime to estimate their probabilities are exponential in N, this is compensated by the fact that there are only exponentially small number of them, and hence the overall runtime of the algorithm can still stay efficient. Because the rest of the outcomes only occur with a near-zero probability, ignoring them doesn't necessarily harm the precision of the algorithm.

If the anti-concentration condition in Conjecture 5 in the main text is true, then we avoid the above scenario. To see this, we start from anti-concentration condition on $\tilde{p}_{\boldsymbol{n}}$ in the main text:

$$\Pr_{\boldsymbol{n}\in\Omega}\left(\tilde{p}_{\boldsymbol{n}} \le \frac{1}{\alpha|\Omega|}\right) < \eta. \tag{S45}$$

The sampling space is assumed to be over the collisionless outcomes and for $N \leq \sqrt{M}$, hence $|\Omega| = \frac{1}{\binom{M}{N}} < \frac{3N!}{2M^N}$. Using the 'hiding' property of the unitary submatrices, the $\tilde{p}_{\boldsymbol{n}}$ function has a distribution of

$$\tilde{p}_{\boldsymbol{n}} \sim \frac{2^N}{F_N(w)M^N} \left|\mathrm{lHaf}\left(\boldsymbol{X}\boldsymbol{X}^T, w\boldsymbol{X}\boldsymbol{1}_N\right)\right|^2 \tag{S46}$$

for $\boldsymbol{X} \in \mathcal{G}_{N,N}(0,1)$, where we rescaled the variance of the Gaussian distribution. Combining Equations S45 and S46 gives the anti-concentration condition in Conjecture 5 in the main text, which is formulated as

$$\Pr_{\boldsymbol{X}\in\mathcal{G}_{N,N}(0,1)}\left[\sqrt{\frac{2^N}{N!F_N(w)}}\left|\mathrm{lHaf}\left(\boldsymbol{X}\boldsymbol{X}^T, w\boldsymbol{X}\boldsymbol{1}_N\right)\right| < \frac{1}{\alpha}\right] < \eta, \tag{S47}$$

and the conjecture is that $\alpha = \mathrm{poly}(N, 1/\eta)$.

The left-hand side of Equation S47 can be considered as some cumulative distribution function (CDF) of the variable

$$\sqrt{\frac{2^N}{N!F_N(w)}}\left|\mathrm{lHaf}\left(\boldsymbol{X}\boldsymbol{X}^T, w\boldsymbol{X}\boldsymbol{1}_N\right)\right|$$

over the Gaussian distribution of matrix $\boldsymbol{X}$. We denote this CDF as $F_{\mathrm{lHaf}(w)}\left(N, \frac{1}{\alpha}\right)$:

$$F_{\mathrm{lHaf}(w)}\left(N, \frac{1}{\alpha}\right) = \Pr_{\boldsymbol{X}\in\mathcal{G}_{N,N}(0,1)}\left[\sqrt{\frac{2^N}{N!F_N(w)}}\left|\mathrm{lHaf}\left(\boldsymbol{X}\boldsymbol{X}^T, w\boldsymbol{X}\boldsymbol{1}_N\right)\right| < \frac{1}{\alpha}\right]. \tag{S48}$$

We can define similar functions for the determinant, the permanent and the Hafnian:

$$F_{\mathrm{det}}\left(N, \frac{1}{\alpha}\right) = \Pr_{\boldsymbol{X}\in\mathcal{G}_{N,N}(0,1)}\left[\sqrt{\frac{1}{N!}}\left|\det\left(\boldsymbol{X}\right)\right| < \frac{1}{\alpha}\right], \tag{S49}$$

$$F_{\mathrm{Per}}\left(N, \frac{1}{\alpha}\right) = \Pr_{\boldsymbol{X}\in\mathcal{G}_{N,N}(0,1)}\left[\sqrt{\frac{1}{N!}}\left|\mathrm{Per}\left(\boldsymbol{X}\right)\right| < \frac{1}{\alpha}\right], \tag{S50}$$

$$F_{\mathrm{Haf}}\left(N, \frac{1}{\alpha}\right) = \Pr_{\boldsymbol{X}\in\mathcal{G}_{N,N}(0,1)}\left[\sqrt{\frac{1}{N!\binom{N-1}{N/2}}}\left|\mathrm{Haf}\left(\boldsymbol{X}\boldsymbol{X}^T\right)\right| < \frac{1}{\alpha}\right]. \tag{S51}$$

Ref. [13] proved that the determinant of Gaussian matrices satisfies the anti-concentration condition. In other words, there exists a polynomial $\alpha = \mathrm{poly}(N, 1/\eta)$, such that for all positive N and real $\eta > 0$:

$$F_{\mathrm{det}}\left(N, \frac{1}{\alpha}\right) < \eta. \tag{S52}$$

Ref. [13] also conjectured that the same is true for the Permanent of Gaussian matrices.

We numerically calculated each function (Equation S48-S51) for 100,000 random matrices $\boldsymbol{X} \in \mathcal{G}_{N,N}(0,1)$ up to $N = 28$ ($N = 26$ for the Permanent). Then, for a fixed η, we estimate the value α for which the CDF satisfies $F\left(N, \frac{1}{\alpha}\right) < \eta$. The results are plotted in Fig. S2.

The scaling of α with N for the loop-Hafnian at $w = 0.1$ looks qualitatively similar to the rest of the functions. This gives reason to believe that the output of Uniform D-GBS is probably at least as anti-concentrated as regular GBS when displacement is low. As the displacement to squeezing ratio increases, α starts increasing faster, but they could still be polynomially bounded at higher N values. Our calculations do not seem to unveil any qualitative differences in the scaling between the Hafnian and the loop-Hafnian with, for example, $w = 0.4$. A value of $w = 0.4$ corresponds to $\sinh^2(r)/|\beta|^2 = 1$, i.e. the mean photon number from squeezing being equal to that from displacement. However, admittedly, due to the exponential scaling of the run-time complexity of computing the loop-Hafnian, we were only able to numerically compute them up to a limited N [21].

As $|w|$ continues to increase, we expect a transition in the anti-concentration properties of the loop-Hafnian as well. In the limiting case where squeezing is reduced to zero, the output probabilities of Uniform D-GBS is given by

$$p_{\boldsymbol{n}} = \mathrm{e}^{-K|\beta|^2}\left|\mathrm{lHaf}\left(\boldsymbol{0}, \boldsymbol{\gamma}_{\boldsymbol{n}}\right)\right|^2 \tag{S53}$$

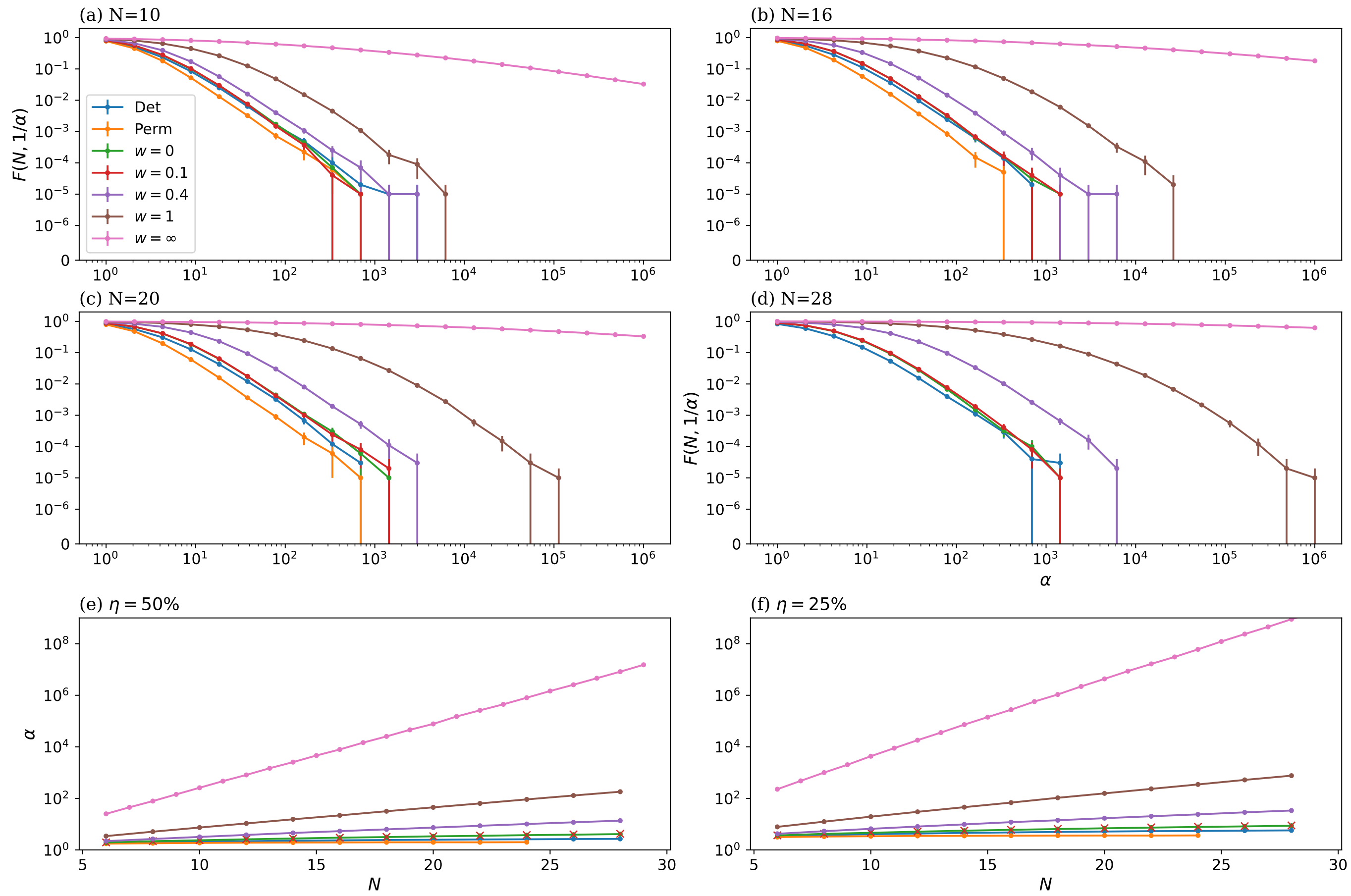

FIG. S2. Numerical estimation of the CDF of various functions over random Gaussian matrices with varying dimensions, for (a) $N = 10$, (b) $N = 16$, (c) $N = 20$, (d) $N = 28$. The relevant functions are calculated over 100,000 random matrices $\boldsymbol{X} \in \mathcal{G}_{N,N}(0,1)$ and the CDF is estimated from these data. Errorbars are estimated from bootstrapping, though for most data points the errorbars are not visible. Legend $w = 0$ represents data for the Hafnian, and legend $w = \infty$ represents data for Equation S56 when only coherent states are used as input for D-GBS. The upper bound α is plotted in (e) for $\eta = 50\%$ and (f) for $\eta = 25\%$. The loop-Hafnian data for $w = 0.1$ are plotted in red crosses as they completely overlap with the Hafnian data.

for $\gamma_i = \beta^* \sum_{j=1}^{K} U_{ij}$. The probability of measuring N photons becomes Poissonian (Equation S18):

$$p_N = \mathrm{e}^{-K|\beta|^2} \frac{\left(K|\beta|^2\right)^N}{N!}. \tag{S54}$$

Combining these equations, the post-selected probability $\tilde{p}_{\boldsymbol{N}}$ in Equation S45 becomes:

$$\tilde{p}_{\boldsymbol{n}} = \frac{N!}{K^N} \prod_{n_i \neq 0} \left| \sum_{j=1}^{K} U_{ij} \right|^2 . \tag{S55}$$

Using the 'hiding' property of the unitary submatrices, the anti-concentration inequality for $\tilde{p}_{\boldsymbol{n}}$ in Equation S45 becomes

$$\Pr_{\boldsymbol{X} \in \mathcal{G}_{N,K}(0,1)} \left[\prod_{i=1}^{N} \left| \sum_{j=1}^{K} \frac{X_{ij}}{\sqrt{K}} \right|^2 < \frac{1}{\alpha} \right] = \Pr_{x_i, y_i \in \mathcal{N}(0, \frac{1}{2})} \left[\prod_{i=1}^{N} (x_i^2 + y_i^2) < \frac{1}{\alpha} \right] < \eta, \tag{S56}$$

where we used the fact that the sum of i.i.d. Gaussian random variables are still a Gaussian distribution. The notation $\mathcal{N}(\mu, \sigma^2)$ denotes the real Gaussian distribution with mean μ and variance σ^2. The distribution of $x_i^2 + y_i^2$ obeys an exponential distribution. Thus the scaling of α has to be at least exponential.

The numerical results provides some level of evidence for believing the loop-Hafnian anti-concentration conjecture to hold up to $w = 0.1$ and maybe even $w = 0.4$. Nevertheless, a definite proof for it remains elusive, as is that in Fock-state Boson Sampling and regular GBS without displacement [13, 15]. We leave for future work to fill this gap. Either verifying our conjecture or invalidating it would profoundly deepen our understanding of the complexity of D-GBS.

It's also worth noting that anti-concentration is not a *necessary* condition for D-GBS to be hard. For example, Ref. [6] provided a complexity argument for regular GBS to be classically hard without explicitly invoking an anti-concentration conjecture. As such, the argument relies on conjecturing that additive-error approximation to the Hafnian is average-case #P-hard. To do so in D-GBS requires similarly arguing that additive-error approximation of the loop-Hafnian is average-case #P-hard for some range of w.

SV. MODELLING A NOISY CHANNEL AS RANDOM DISPLACEMENTS

In a realistic GBS experiment, the Gaussian state is subject to a noisy channel, with transmission η. For a zero-mean state, this implements the map: $\mathbf{\Sigma} \mapsto \mathbf{\Sigma}' = \eta\mathbf{\Sigma} + \frac{1}{2}(1-\eta)\mathbb{1}_{2M}$, which is a mixed state.

As described in [28, 30], the covariance matrix can therefore be written as $\mathbf{\Sigma}' = \boldsymbol{T} + \boldsymbol{W}$, in which $\boldsymbol{T}$ is a pure state (with a lower average photon number than $\mathbf{\Sigma}'$), and $\boldsymbol{W} \geq 0$ is the classical Gaussian noise. This can be simulated by considering a state with covariance matrix $\boldsymbol{T}$, and displacement drawn randomly from the multivariate Gaussian distribution:

$$p(\boldsymbol{\mu}) = \frac{\exp(-(1/2)\boldsymbol{\mu}^T \boldsymbol{W}^{-1}\boldsymbol{\mu})}{\sqrt{\det(2\pi\boldsymbol{W})}}. \tag{S57}$$

In Ref. [27], this is utilised to reduce the time of simulation of GBS, as introducing a displacement can be operated locally and does not increase the complexity of tensor network methods.

In the main text, we consider that for a sufficiently high displacement/squeezing ratio, efficient classical algorithms are likely to succeed in simulating D-GBS. For a zero-mean state undergoing a lossy channel, if the loss exceeds a certain threshold, the above method can be used, in which case the majority of samples will have a displacement/squeezing ratio above what is required for efficient classical simulation.

The decomposition $\mathbf{\Sigma} = \boldsymbol{T} + \boldsymbol{W}$ is non-unique. As described in Ref. [27], this can be optimised to minimise the average photon number of $\boldsymbol{T}$ using semidefinite programming. In this section, we consider an analytic decomposition for an arbitrary state using the Williamson decomposition [31, 32], $\mathbf{\Sigma} = \boldsymbol{S}\boldsymbol{D}\boldsymbol{S}^\dagger$, in which $\boldsymbol{S}$ is a symplectic matrix and $\boldsymbol{D}$ is a diagonal matrix ($\boldsymbol{D} = \frac{1}{2}\mathbb{1}$ when $\mathbf{\Sigma}$ represents a pure state). We note that this was done in Ref. [27] for a single-mode squeezed state.

We consider $\boldsymbol{\sigma}' = \eta\boldsymbol{\sigma} + (1-\eta)\frac{1}{2}\mathbb{1}_{2M}$ in which $\boldsymbol{\sigma}$ is expressed in the real basis, that is, using the basis vector $\hat{\boldsymbol{r}} = (\hat{x}_1, ..., \hat{x}_M, \hat{p}_1, ..., \hat{p}_M)^T$. We first consider the multi-mode squeezed state, with $\boldsymbol{\sigma} = \frac{1}{2}\mathrm{diag}(\mathrm{e}^{-2r}, ..., \mathrm{e}^{-2r}, \mathrm{e}^{2r}, ..., \mathrm{e}^{2r})$, where all modes are squeezed with the same parameter r. As described in, e.g. Ref. [33], the symplectic eigenvalues are given by the positive eigenvalues of $i\boldsymbol{\Omega}\boldsymbol{\sigma}'$, which are $\lambda = \sqrt{\frac{1}{4} + (\eta - \eta^2)\sinh^2(r)}$. The matrix $\boldsymbol{\Omega}$ is defined as $\boldsymbol{\Omega} = \begin{pmatrix} 0 & \mathbb{1}_M \\ -\mathbb{1}_M & 0 \end{pmatrix}$.

Given that $\boldsymbol{\sigma}' = \boldsymbol{S}\boldsymbol{D}\boldsymbol{S}^T$, we can find $\boldsymbol{S}$ by using the decomposition

$$\boldsymbol{S} = \boldsymbol{D}^{-1/2}\boldsymbol{U}^T(\boldsymbol{\sigma}')^{1/2} = \boldsymbol{U}^T \mathrm{diag}(\mathrm{e}^{-r'}, ..., \mathrm{e}^{-r'}, \mathrm{e}^{r'}, ..., \mathrm{e}^{r'}), \tag{S58}$$

where r' is the squeezing parameter of the pure state part represented by $\boldsymbol{T}$, and $\boldsymbol{U} := \sqrt{2}(\boldsymbol{u}_1, ..., \boldsymbol{u}_m, \boldsymbol{v}_1, ..., \boldsymbol{v}_m)$, where $\boldsymbol{u}_i + i\boldsymbol{v}_i$ are the eigenvectors of $(\boldsymbol{\sigma}')^{1/2}\boldsymbol{\Omega}(\boldsymbol{\sigma}')^{1/2}$. Hence we find that the new squeezing value is

$$\begin{aligned} r' &= \ln\left(\frac{1}{\sqrt{2}}\lambda^{-1/2}(\eta\mathrm{e}^{2r} + 1 - \eta)^{1/2}\right) \\ &= \frac{1}{4}\ln\left(\frac{\eta\mathrm{e}^{2r} + 1 - \eta}{\eta\mathrm{e}^{-2r} + 1 - \eta}\right), \end{aligned} \tag{S59}$$

in agreement with [27]. Intuitively we would expect that passing the state through an interferometer should not change the magnitude of squeezing and displacement. Indeed, in the decomposition, this should just be equivalent to just changing U, which does not change the squeezing value.

We now find that the classical Gaussian noise part is represented by

$$\begin{aligned} \boldsymbol{W} &= \boldsymbol{S}(\boldsymbol{D} - \frac{1}{2}\mathbb{1})\boldsymbol{S}^T \\ &= (\lambda - \frac{1}{2})\boldsymbol{S}\boldsymbol{S}^T, \end{aligned} \tag{S60}$$

with $\boldsymbol{S} = \frac{1}{\sqrt{\lambda}}\boldsymbol{U}^T(\boldsymbol{\sigma}')^{1/2}$. We find that $\boldsymbol{U} = \begin{pmatrix} \mathbb{1} & 0 \\ 0 & -\mathbb{1} \end{pmatrix}$, and so $\boldsymbol{W} = (1 - \frac{1}{2\lambda})\boldsymbol{\sigma}'$.

We then draw displacements from Eq. S57. If the real displacement is $\boldsymbol{d}^T = (\boldsymbol{d}_1^T, \boldsymbol{d}_2^T)$, then the complex basis displacement vector is $\boldsymbol{\mu}^T = (\boldsymbol{d}_1^T + i\boldsymbol{d}_2^T, \boldsymbol{d}_1^T - i\boldsymbol{d}_2^T)$. Hence, we apply to each mode a displacement β that is normally distributed with mean 0. The real part has variance $\frac{1}{2}(1 - \frac{1}{2\lambda})(\eta \mathrm{e}^{-2r} + 1 - \eta)$ and the imaginary part has variance $\frac{1}{2}(1 - \frac{1}{2\lambda})(\eta \mathrm{e}^{2r} + 1 - \eta)$.

We are interested in the magnitude of $w = \frac{\beta^* - \beta \tanh(r')}{\sqrt{\tanh(r')}}$,which follows a Hoyt distribution [34]. It is expected that half of the sampled displacements have $|w|$ less than or equal to the circular error probable (CEP), which is given by [35]:

$$\mathrm{CEP}(w) = 1.17741 s'(1 - 0.163357\rho'^2 - 0.041694\rho'^4), \tag{S61}$$

where $s' = \frac{1}{2}(\mathrm{STD}(\Re(w))^2 + \mathrm{STD}(\Im(w))^2)$, and $\rho' = (\mathrm{STD}(\Im(w))^2 - \mathrm{STD}(\Re(w))^2)/(\mathrm{STD}(\Re(w))^2 + \mathrm{STD}(\Im(w))^2)$.

Using:

$$\mathrm{STD}(\Re(w)) = \sqrt{\frac{(1 - \tanh(r'))^2}{2\tanh(r')}(1 - \frac{1}{2\lambda})(\eta \mathrm{e}^{-2r} + 1 - \eta)} \tag{S62}$$

$$\mathrm{STD}(\Im(w)) = \sqrt{\frac{(1 + \tanh(r'))^2}{2\tanh(r')}(1 - \frac{1}{2\lambda})(\eta \mathrm{e}^{2r} + 1 - \eta)}, \tag{S63}$$

we find that:

$$\begin{aligned} \rho' &= \frac{(1 + \tanh(r'))^2(\eta \mathrm{e}^{2r} + 1 - \eta) - (1 - \tanh(r'))^2(\eta \mathrm{e}^{-2r} + 1 - \eta)}{(1 + \tanh(r'))^2(\eta \mathrm{e}^{2r} + 1 - \eta) + (1 - \tanh(r'))^2(\eta \mathrm{e}^{-2r} + 1 - \eta)} \\ &= \frac{(\eta \mathrm{e}^{2r} + 1 - \eta)^2 - (\eta \mathrm{e}^{-2r} + 1 - \eta)^2}{(\eta \mathrm{e}^{2r} + 1 - \eta)^2 + (\eta \mathrm{e}^{-2r} + 1 - \eta)^2} \\ &= \frac{4\eta \sinh(r)\cosh(r)(2\eta \sinh^2(r) + 1)}{4\eta^2(2\sinh^4(r) + \sinh^2(r)) + 4\eta \sinh^2(r) + 1} \end{aligned} \tag{S64}$$

and:

$$\begin{aligned} s' &= \frac{1 - (1/2\lambda)}{4\tanh(r')}((1 + \tanh(r'))^2(\eta \mathrm{e}^{2r} + 1 - \eta) + (1 - \tanh(r'))^2(\eta \mathrm{e}^{-2r} + 1 - \eta)) \\ &= \frac{\eta^2(4\sinh^2(r) + 8\sinh^4(r)) + 4\eta \sinh^2(r) + 1}{2\eta \sinh(r)\cosh(r)}(1 - 1/\sqrt{4\eta \sinh^2(r)(1 - \eta) + 1}) \end{aligned} \tag{S65}$$

Using Eq. S61, we can see how the CEP of w varies based on transmission, η, and the initial squeezing parameter, r. However, we are more concerned with the amount of loss we can tolerate as we scale up the expected photon number from the sources, $\bar{N} = K\sinh^2(r)$. We note that in this construction, $K = M$, and we assume that r is chosen so that $\bar{N} = \sqrt{M}$, in which case $\sinh^2(r) = \bar{N}^{-1}$. We consider the proportion of surviving photons to be $\eta \bar{N} = \bar{N}^{\alpha}$.

We have

$$\sinh^2(r) = \frac{1}{\bar{N}},\ \cosh^2(r) = \left(1 + \frac{1}{\bar{N}}\right),\ \sinh(r)\cosh(r) = \frac{\sqrt{\bar{N} + 1}}{N},\ \eta = \bar{N}^{\alpha - 1}. \tag{S66}$$

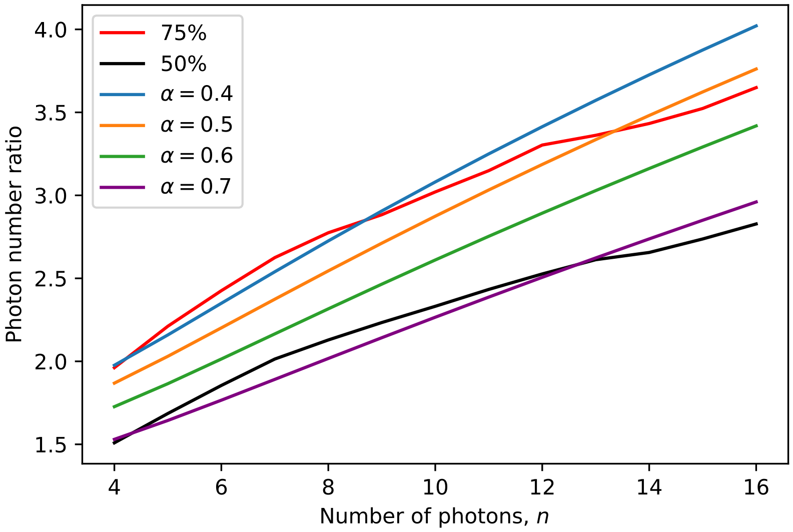

FIG. S3. Here, we plot the photon number ratio that corresponds to the median value of $|w|$, when approximating lossy Gaussian states using random displacements. The different values of α parametrise loss levels. We also include the numerical results presented in Fig. 5 for comparison, showing the values of $|w|$ for which the simulation method has a 50% or 75% chance to be successful.

Subbing into ρ' gives us

$$\begin{aligned}\rho' &= \frac{4\eta\sqrt{\bar{N}+1}\bar{N}^{-1}\left(2\eta\bar{N}^{-1}+1\right)}{4\eta^2\left(2\bar{N}^{-2}+\bar{N}^{-1}\right)+4\eta\bar{N}^{-1}+1} \\ &= \frac{4\left(\bar{N}+1\right)^{\frac{1}{2}}\bar{N}^{\alpha-2}\left(2\bar{N}^{\alpha-2}+1\right)}{4\bar{N}^{2(\alpha-1)}\left(2\bar{N}^{-2}+\bar{N}^{-1}\right)+4\bar{N}^{\alpha-2}+1}\end{aligned} \tag{S67}$$

$$\begin{aligned}s' &= \frac{\eta^2\left(8\bar{N}^{-2}+4\bar{N}^{-1}\right)+4\eta\bar{N}^{-1}+1}{2\eta\left(\bar{N}+1\right)^{\frac{1}{2}}\bar{N}^{-1}}\left(1-\frac{1}{\sqrt{4\eta(1-\eta)\bar{N}^{-1}+1}}\right) \\ &= \frac{\bar{N}^{2(\alpha-1)}\left(8\bar{N}^{-2}+4\bar{N}^{-1}\right)+4\bar{N}^{\alpha-2}+1}{2\left(\bar{N}+1\right)^{\frac{1}{2}}\bar{N}^{\alpha-2}}\left(1-\frac{1}{\sqrt{4(1-\bar{N}^{\alpha-1})\bar{N}^{\alpha-2}+1}}\right)\end{aligned} \tag{S68}$$

The CEP of w can be thought of as representing the median value of $|w|$ across many samples. It is still not clear on the growth of $|w|$ with increasing photon number that it is required in order for the efficient classical algorithms described in this work (or others) to be applicable. However, it is clear from our considerations of D-GBS that $|w|$ must be at least constant with increasing $\bar{N}$.

In Fig. S3, we compare the median value of $|w|$ for several values of α to the numerical results generated for the expected values required for 50% or 75% of matrices to be simulable. That is, we expect that above these loss values, for the majority of samples, the efficient simulation methods will be effective the majority (more than half) of the time.

These results do not seem to improve on currently understood loss thresholds of $\alpha = 0.5$. Nonetheless, the closeness of these results, and the fact that the lines corresponding to simulation of lossy GBS seem to be less steep than the numerical results, indicating that for higher values of N, lower loss values may be necessary for the simulation methods we have proposed to be effective.

Furthermore, the analytic decomposition used is not optimal. As described in Ref. [27], semi-definite programming can be used to optimise the decomposition to minimise the photon number of the pure state T. In this case, the efficient classical algorithms we have described may be applicable above a lower loss threshold.

We also note that efficient classical methods to simulate GBS with high amounts of loss would not necessarily work to simulate D-GBS using this method, due to the fact that the displacement is not fixed but chosen randomly across samples.

[1] R. Simon, N. Mukunda, and B. Dutta, Quantum-noise matrix for multimode systems: U(n) invariance, squeezing, and normal forms, Phys. Rev. A **49**, 1567 (1994).
[2] R. Kruse, C. S. Hamilton, L. Sansoni, S. Barkhofen, C. Silberhorn, and I. Jex, Detailed study of Gaussian boson sampling, Phys. Rev. A **100**, 032326 (2019).
[3] N. Quesada, L. G. Helt, J. Izaac, J. M. Arrazola, R. Shahrokhshahi, C. R. Myers, and K. K. Sabapathy, Simulating realistic non-Gaussian state preparation, Phys. Rev. A **100**, 022341 (2019).
[4] S. Wang, H.-Y. Fan, and L.-Y. Hu, Photon-number distributions of non-Gaussian states generated by photon subtraction and addition, J. Opt. Soc. Am. B **29**, 1020 (2012).
[5] E. Fitzke, F. Niederschuh, and T. Walther, Simulating the photon statistics of multimode Gaussian states by automatic differentiation of generating functions, APL Photonics **8**, 10.1063/5.0129638 (2023).
[6] A. Deshpande, A. Mehta, T. Vincent, N. Quesada, M. Hinsche, M. Ioannou, L. Madsen, J. Lavoie, H. Qi, J. Eisert, D. Hangleiter, B. Fefferman, and I. Dhand, Quantum computational advantage via high-dimensional Gaussian boson sampling, Science Advances **8**, eabi7894 (2022).
[7] R. Loudon, *The quantum theory of light* (OUP Oxford, 2000).
[8] W. Feller, *An introduction to probability theory and its applications, Volume 2*, Vol. 2 (John Wiley & Sons, 1991).
[9] R. Kotecký and D. Preiss, Cluster expansion for abstract polymer models, Commun. Math. Phys. **103**, 491 (1986).
[10] A. Barvinok, Computing permanents of complex diagonally dominant matrices and tensors (2018), arXiv:1801.04191 [math.CO].
[11] M. Jerrum, Two-dimensional monomer-dimer systems are computationally intractable, J. Stat. Phys. **48**, 121 (1987).
[12] I. Bezakova, A. Galanis, L. A. Goldberg, and D. Stefankovic, The complexity of approximating the matching polynomial in the complex plane (2021), arXiv:1807.04930 [cs.DM].
[13] S. Aaronson and A. Arkhipov, The computational complexity of linear optics, in *Proceedings of the Forty-Third Annual ACM Symposium on Theory of Computing*, STOC '11 (Association for Computing Machinery, New York, NY, USA, 2011) p. 333–342.
[14] L. Stockmeyer, The complexity of approximate counting, in *Proceedings of the Fifteenth Annual ACM Symposium on Theory of Computing*, STOC '83 (Association for Computing Machinery, New York, NY, USA, 1983) p. 118–126.
[15] D. Hangleiter and J. Eisert, Computational advantage of quantum random sampling, Rev. Mod. Phys. **95**, 035001 (2023).
[16] M. J. Bremner, A. Montanaro, and D. J. Shepherd, Average-case complexity versus approximate simulation of commuting quantum computations, Phys. Rev. Lett. **117**, 080501 (2016).
[17] R. Movassagh, The hardness of random quantum circuits, Nature Physics **19**, 1719 (2023).
[18] R. J. Lipton, New directions in testing, in *Distributed computing and cryptography*, Vol. 2, edited by J. Feigenbaum and M. J. Merritt (American Mathematical Society, 1991) pp. 191–202.
[19] A. Bouland, B. Fefferman, Z. Landau, and Y. Liu, Noise and the frontier of quantum supremacy, in *2021 IEEE 62nd Annual Symposium on Foundations of Computer Science (FOCS)* (2022) pp. 1308–1317.
[20] E. R. Caianiello, On quantum field theory — I: explicit solution of Dyson's equation in electrodynamics without use of feynman graphs, Il Nuovo Cimento (1943-1954) **10**, 1634 (1953).
[21] J. F. F. Bulmer, B. A. Bell, R. S. Chadwick, A. E. Jones, D. Moise, A. Rigazzi, J. Thorbecke, U.-U. Haus, T. V. Vaerenbergh, R. B. Patel, I. A. Walmsley, and A. Laing, The boundary for quantum advantage in Gaussian boson sampling, Science Advances **8**, eabl9236 (2022).
[22] H.-S. Zhong, H. Wang, Y.-H. Deng, M.-C. Chen, L.-C. Peng, *et al.*, Quantum computational advantage using photons, Science **370**, 1460 (2020).
[23] H.-S. Zhong, Y.-H. Deng, J. Qin, H. Wang, M.-C. Chen, *et al.*, Phase-programmable Gaussian Boson Sampling using stimulated squeezed light, Phys. Rev. Lett. **127**, 180502 (2021).
[24] Y.-H. Deng, Y.-C. Gu, H.-L. Liu, S.-Q. Gong, H. Su, *et al.*, Gaussian Boson Sampling with pseudo-photon-number-resolving detectors and quantum computational advantage, Phys. Rev. Lett. **131**, 150601 (2023).
[25] L. S. Madsen, F. Laudenbach, M. Falamarzi. Askarani, F. Rortais, T. Vincent, *et al.*, Quantum computational advantage with a programmable photonic processor, Nature **606**, 75 (2022).
[26] G. S. Thekkadath, S. Sempere-Llagostera, B. A. Bell, R. B. Patel, M. S. Kim, and I. A. Walmsley, Experimental demonstration of Gaussian Boson Sampling with displacement, PRX Quantum **3**, 020336 (2022).
[27] C. Oh, M. Liu, Y. Alexeev, B. Fefferman, and L. Jiang, Classical algorithm for simulating experimental Gaussian boson sampling, Nat. Phys. , 1 (2024).
[28] N. Quesada, R. S. Chadwick, B. A. Bell, J. M. Arrazola, T. Vincent, H. Qi, and R. García-Patrón, Quadratic speed-up for simulating Gaussian Boson Sampling, PRX Quantum **3**, 010306 (2022).
[29] S. Rahimi-Keshari, T. C. Ralph, and C. M. Caves, Sufficient conditions for efficient classical simulation of quantum optics, Phys. Rev. X **6**, 021039 (2016).
[30] A. Serafini, *Quantum continuous variables: a primer of theoretical methods* (CRC press, 2017).
[31] J. Williamson, On the algebraic problem concerning the normal forms of linear dynamical systems, American journal of mathematics **58**, 141 (1936).
[32] M. A. De Gosson, *Symplectic geometry and quantum mechanics*, Vol. 166 (Springer Science & Business Media, 2006).
[33] G. Adesso, S. Ragy, and A. R. Lee, Continuous variable quantum information: Gaussian states and beyond, Open Systems & Information Dynamics **21**, 1440001 (2014).

[34] R. Myers, *Data management and statistical analysis techniques* (Scientific e-Resources, 2019).
[35] J. Krempasky, CEP equation exact to the fourth order, Navigation **50**, 143 (2003).
